# Interfacial Nanoengineering of Hydrogel Surfaces via Block Copolymer Self-Assembly

**DOI:** 10.1021/acsami.4c18632

**Published:** 2025-02-04

**Authors:** Andrea Cosimi, Daniel D. Stöbener, Philip Nickl, Robert Schusterbauer, Ievgen S. Donskyi, Marie Weinhart

**Affiliations:** † Freie Universität Berlin, Institute of Chemistry and Biochemistry − Organic Chemistry, Takustraße 3, Berlin 14195, Germany; ‡ Leibniz Universität Hannover, Institute of Physical Chemistry and Electrochemistry, Callinstraße 3A, Hannover 30167, Germany; § BAM − Federal Institute for Material Science and Testing − Division of Surface Analytics, and Interfacial Chemistry, Unter den Eichen 44-46, Berlin 12205, Germany

**Keywords:** brushing-up, benzophenone, LCST-type polymer, poly(glycidyl ether) (PGE), fibroblast adhesion

## Abstract

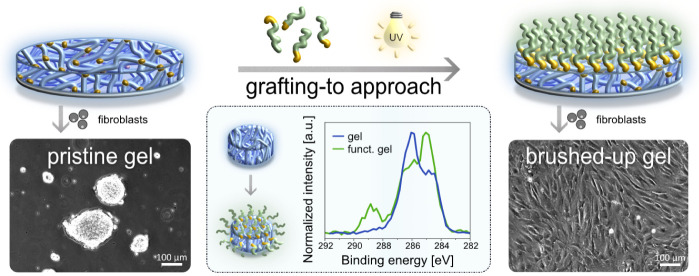

Synthetic polymer hydrogels are valuable matrices for biotransformations, drug delivery, and soft implants. While the bulk properties of hydrogels depend on chemical composition and network structure, the critical role of interfacial features is often underestimated. This work presents a nanoscale modification of the gel–water interface using polymer brushes via a straightforward “grafting-to” strategy, offering an alternative to more cumbersome “grafting-from” approaches. Functional block copolymers with photoreactive anchor blocks are successfully self-assembled and UV-immobilized on hydrogel substrates despite their low solid content (<30 wt %). This versatile technique works on both bulk- and surface-immobilized hydrogels, demonstrated on poly(hydroxypropyl acrylate), poly(*N*-isopropylacrylamide), and alginate gels, allowing precise control over grafting density. X-ray photoelectron spectroscopy and time-of-flight secondary ion mass spectrometry revealed a homogeneous bilayered architecture. By “brushing-up”, the hydrogels’ interface can be tailored to enhance protein adsorption, improve cell adhesion, or impair the diffusive uptake of small molecules into the bulk gels. This effective interfacial nanoengineering method is broadly applicable for enhancing hydrogel performance across a wide range of applications.

## Introduction

The engineering of interfacial properties of solid surfaces is a common strategy to control wetting, adhesion, friction, and/or the biological response at the material surface.^1^ Therefore, manifold approaches for solid substrate modification have been established, including polishing, plasma treatment, thin film coatings, and surface grafting techniques.^2^ Generally, the covalent attachment of functional polymer brushes on the substrate surface is accomplished by “grafting-from” and “grafting-to” strategies.^3,4^ In the “grafting-from” approach, polymer brush formation is achieved through surface-initiated polymerization (SIP) from specifically premodified initiating sites on the substrate surface,^5,6^ enabling very high grafting densities (GDs) up to >1 chain/nm.^2,7^ In contrast, complementary “grafting-to” approaches utilize preformed polymers with reactive end groups or anchor blocks to functionalize the substrate’s surface, typically achieving limited GDs up to 0.5 chains/nm^2^ due to the steric repulsion of the assembling chains. However, the primary advantage of the “grafting-to” over the “grafting-from” approach, despite its GD limitations, lies in the ability to produce molecularly defined functional coatings with precise control over polymer molecular weight, dispersity, architecture, and microstructure.^8^ In comparison, even with advanced surface analysis techniques, the molecular characteristics of polymer brushes prepared via “grafting-from” methods often remain obscure.^9,10^ Both techniques, however, are well-established for use on a wide range of solid substrates, including hard and soft materials, as well as porous solids.^6,11^

In contrast, water-swollen hydrogels have surprisingly received little attention regarding functional engineering of their interfacial properties. While numerous synthetic strategies have been developed to enhance and tailor the functional features of hydrogels, these have primarily focused on controlling their composition and internal microstructure.^12^ “Smart” features, by employing, e.g., thermoresponsive polymers with a lower critical solution temperature (LCST), enable temporal control over the hydrophilicity and mesh size of the gels.^13,14^ The thermally induced, reversible conformational chain rearrangement within a thermoresponsive gel, triggered at the volume phase transition temperature, facilitates transitions between swollen and collapsed state, offering on-demand control over the gel’s physicochemical properties.^15^ For instance, thermally controlled protein adsorption on the surface can influence and guide mammalian cell attachment and proliferation,^16^ thereby directly affecting the hydrogel’s performance and fate in biological environments.

Functionalizing the hydrogel surface with polymer brushes adds complementary functional features, such as tunable adhesion,^5,17^ friction,^18−20^ or permeability,^21^ expanding the material’s potential applications. “Brushing up” soft, water-swollen hydrogel surfaces by polymer brush grafting poses significant challenges due to their high water content and relatively low solid fraction, which rarely exceeds 30 wt %.^11^ Additionally, solvent removal often leads to the formation of highly porous structures. To date, only “grafting-from” techniques based on SIP have been successfully employed to grow polymer brushes on hydrogel surfaces. Controlled radical polymerizations are the method of choice since they are compatible with water.^22^ In contrast, alternative “grafting-to” methods allow the attachment of preformed polymers synthesized under both water-free and aqueous conditions, overcoming the limitation of “grafting-from” techniques on hydrogels. Moreover, the “grafting-to” approach eliminates residual chemicals or catalyst traces from SIP, reducing cytotoxicity risks and enhancing the suitability of brushed-up hydrogels for biomedical applications.^6^

Based on our experience with the directed self-assembly of thermoresponsive poly(glycidyl ether) (PGE) block copolymers on glass and plastic substrates,^23,24^ we propose their use for nanoengineering soft and swollen hydrogel interfaces via a “grafting-to” approach. We have shown that short benzophenone (BP)-based anchor blocks containing amide groups can drive selective adsorption of block copolymers on hydrophilized plastic substrates through combined hydrogen bonding, amide−π interactions, and Van der Waals forces.^25^ We hypothesize that similar interactions can drive effective block copolymer self-assembly on hydrophilic, swollen hydrogels. As a biocompatible type II photoinitiator, BP further enables the covalent attachment of the assembled polymer brushes to an underlying substrate upon brief UV irradiation (λ ∼ 350–365 nm).^26^ Compared to highly reactive carbene and nitrene intermediate-forming photoactive cross-linkers,^27−30^ carbon radical-forming BP inserts selectively into C–H bonds followed by C–C bond formation. Furthermore, with growing regulatory restrictions on the use of polyfluorinated compounds, BP-based photografting offers a promising fluorine-free alternative to the effective perfluorophenyl azide photo-cross-linking.^29,30^

In this pioneering work, we introduce a versatile “grafting-to” strategy for surface-bound and bulk hydrogel interfaces, allowing precise control over the GD of brushed-up structures, hereafter referred to as bilayers. We successfully transferred the protocol for the self-assembly of PGE block copolymers (PGE-*block*-BP), featuring photoreactive BP anchor blocks, from hard substrates to soft hydrogels with solid contents as low as 26 wt %. Impressively, both block copolymer assembly and subsequent UV-induced covalent immobilization were effectively achieved on the solvated, swollen interface. The functional impact of these nanoengineered hydrogel interfaces was demonstrated by drastically enhanced protein adsorption and cell adhesiveness, enabling the thermally triggered detachment of confluent cell sheets as well as altered diffusive barrier properties at the gel interface.

## Experimental Section

Supporting Information provides a detailed list of materials and methods. The photoreactive monomer 4-acryloyloxybenzophenone (4-ABP) was synthesized according to a modified procedure of Zhang et al.,^31^ which is described briefly in the Supporting Information, including characterization.

### Materials for Synthesis

Hydroxypropyl acrylate (HPA) was purchased from TCI Deutschland GmbH (Eschborn, Germany) as a mixture of 2-hydroxypropyl acrylate (2-HPA) and 1-methyl-2-hydroxyethyl acrylate (1-MeHEA) and used after filtration through Al_2_O_3_ to remove hydroquinone monomethyl ether as the inhibitor. 4-Acryloyloxybenzophenone (4-ABP) was synthesized according to Scheme S1. *N*-isopropyl acrylamide (NIPAm) and 2,2′-azobis(2-methylpropionitrile) (AIBN, 98%) were supplied by Sigma-Aldrich (Steinheim, Germany) and used after recrystallization in *n*-pentane:*n*-hexane (6:4) for NIPAm and methanol (MeOH, ≥99.9%) for AIBN. *N*,*N*′-methylene bis(acrylamide) (MBAA) and MeOH for dialysis of polymers were purchased from Sigma-Aldrich and used without further purification. Dichloromethane (DCM) was supplied by Fisher Scientific (Schwerte, Germany). Sodium sulfate (Na_2_SO_4_, 99%) and prewetted regenerated cellulose dialysis tubes (molecular weight cutoff (MWCO): 3.5 kDa, Spectra/Por 6) from SpectrumLabs were supplied by Carl Roth GmbH + Co. KG (Karlsruhe, Germany). Ethyl acetate (EtOAc) was supplied by Merck (Darmstadt, Germany). Ethanol (EtOH, technical grade) used for the synthesis as well as for surface preparation was supplied by Sigma-Aldrich (Steinheim, Germany) and distilled under reduced pressure prior to use to remove impurities, while polymer stock solutions for the self-assembly process were prepared from absolute ethanol (EtOH_abs_, 99%, Fisher Scientific, Schwerte, Germany). Sodium bicarbonate (NaHCO_3_) was supplied by Grüssing GmbH (Filsum, Germany).

### Materials for Surface Modification and Characterization

Ultrapure water for surface modification and washing was prepared via a Merck Millipore water treatment system, Milli-Q, with a minimum resistivity of 18.2 MΩ·cm (25 °C). For surface characterization, silicon wafers with a 2 nm SiO_2_ layer supplied by Silchem GmbH (Freiberg, Germany) were cut into quadratic pieces (11 × 11 mm), washed with EtOH, and dried under a stream of N_2_. Gold-coated QCM-D sensor chips (11 mm diameter) were supplied by Q-Sense LOT-Quantum Design GmbH (Darmstadt, Germany). The polystyrene (PS) solution (1 wt % in toluene) used for spin-coating of the silicon wafers/gold sensors was prepared using commercial PS (*M*_n_ = 132 g/mol, *Đ* = 1.9) from Falcon culture dishes supplied by Th. Geyer GmbH + Co. KG (Berlin, Germany). PHPA-*stat*-ABP solutions (0.5 wt % for 15 nm coatings and 1.5 wt % for 50 nm coatings) in EtOH were prepared by dilution of a 2 wt % stock solution in EtOH. Similarly, PNIPAm-*stat*-ABP solutions (0.5 wt % for 15 nm coatings and 2 wt % for 100 nm coatings) in EtOH were prepared by dilution of a 2 wt % stock solution in EtOH. Poly(glycidyl ether) (PGE) block copolymers comprising a short anchor block for surface immobilization were synthesized from glycidyl ethers − glycidyl methyl ether (GME), ethyl glycidyl ether (EGE) and allyl glycidyl ether (AGE) − via anionic ring-opening polymerization with tetraoctylammonium bromide (N(Oct_4_)Br) as initiator and triisobutylaluminum (Al(*i*-Bu_3_) as activator.^24^ A two-step postmodification of the short AGE-based block as described previously yielded PGE block copolymer (PGE-*block*-BP) with amide-linked, photoreactive BP units, which serve for self-assembly on surfaces and covalent immobilization.^24^ The 1:3 comonomer ratio of GME and EGE in the statistical PGE block yields thermoresponsive properties of the copolymer in water with a cloud point at ∼20 °C.^32^ For the block copolymer self-assembly, the PGE-*block*-BP (*M*_n_ = 28.1 kDa; *Đ* = 1.18; GME:EGE = 1:3; 5 BP units) solutions were prepared by dissolving the polymer in aq. EtOH (H_2_O:EtOH 48:52 v/v %-). Notably, preparing the PGE-*block*-BP solutions with freshly distilled but technical-grade EtOH (Sigma-Aldrich) led to cloudiness of the solution due to residual water in the EtOH. By employing EtOH_abs_, the solution appeared transparent. Therefore, all aq. PGE-*block*-BP solutions used in this study were prepared with EtOH_abs_. Phosphate-buffered saline (PBS) solutions used for QCM-D analysis were prepared by dissolving PBS pellets (Sigma-Aldrich, Steinheim, Germany) in Milli-Q water. Before use, PBS solutions were sterile filtered (0.22 μm) and degassed in an ultrasonic bath for 30 min. Sodium alginate was purchased from Sigma-Aldrich (#71238). Calcium chloride (97%) and Lucifer Yellow (lithium salt) were purchased from Thermo Fisher Scientific (Darmstadt, Germany).

### Materials for Cell Culture and Protein Adsorption Studies

Falcon PS culture dishes (Ø 35 mm) were purchased from Th. Geyer GmbH + C. KG (Berlin, Germany). Dulbecco’s modified Eagle medium (DMEM) with 4.5 g L^–1^ glucose, 1% penicillin-streptomycin, trypsin/EDTA solution (0.05%), and Dulbecco’s phosphate-buffered saline solution containing CaCl_2_ and MgCl_2_ (DPBS) were purchased from Thermo Fisher Scientific (Darmstadt, Germany). Fetal bovine serum (FBS) was purchased from PAN-Biotech GmbH (Aidenbach, Germany). Propidium iodide (PI) and fluorescein diacetate (FDA) were supplied by Sigma-Aldrich (Steinheim, Germany).

### Synthesis of PHPA-*stat*-ABP

Commercially available HPA was used as the monomer for synthesizing PHPA-*stat*-ABP (Scheme S2). To reach high molecular weights (>30 kDa) via free radical polymerization, a modified procedure of Popescu et al.^33^ was applied. After inhibitor removal, HPA (1.72 g, 13.22 mmol) was placed in a 50 mL one-neck round-bottom flask together with 0.5 mol % AIBN (11 mg, 6.69 × 10^–5^ mol). Subsequently, 4-ABP (68.1 mg, 2.7 × 10^–4^ mmol) was added to the reaction flask and protected from light. Then, EtOH (3.27 mL, 60 wt %) was added to the reaction flask to reduce the viscosity of the polymerization mixture. The reaction mixture was degassed by Ar flushing for 30 min, then placed in a preheated oil bath at 55 °C to initiate the reaction (55 °C was chosen to reduce the probability of termination reactions that limited the molecular weight of the reported PHPA when the reaction was performed at 70 °C) and kept under these conditions while stirring for 20 h in the absence of light. The reaction was quenched by exposure to air, concentrated, and dried under reduced pressure. The crude polymer was dissolved in MeOH and dialyzed against MeOH (MWCO = 3.5 kDa) with a daily solvent exchange for 3 days. After solvent removal, the pure copolymer (*M*_n_ = 51 kDa; *Đ* = 3.04; 2.0 mol % BP incorporated) was obtained as a colorless solid in 86% yield. Detailed characterization (Figure S2) is provided in the Supporting Information.

### Synthesis of PNIPAm-*stat*-ABP

After recrystallization, NIPAm (1.52 g, 13.44 mmol) was placed into a 50 mL one-neck round-bottom flask together with 0.5 mol % AIBN (11.1 mg, 6.75 × 10^–5^ mol), 2 mol % 4-ABP (69.6 mg, 2.74 × 10^–4^ mol), and EtOH (5.78 mL, 75 wt %) with the reaction flask protected from light. The reaction mixture was degassed by Ar flushing for 30 min, placed in a preheated oil bath at 60 °C to initiate the reaction, and kept under these conditions while stirring for 20 h in the absence of light. The reaction was quenched by exposure to air, concentrated, and dried under reduced pressure. The crude polymer was dissolved in MeOH and dialyzed against MeOH (MWCO = 3.5 kDa) with daily solvent exchange for 3 days. After solvent removal, the pure copolymer (*M*_n_ = 62 kDa; *Đ* = 3.63; 2.4 mol % BP incorporated) was obtained as a colorless solid in 88% yield. The reaction scheme (Scheme S3) and characterization (Figure S3) are provided in the Supporting Information.

## Methods for Surface Modification and Characterization

### Spin Coating of Thin Polystyrene (PS) Films and Hydrogel Precursors

For convenient surface characterization, the pristine and brushed-up gel coatings were prepared on thin PS films on silicon substrates and sensors for a quartz crystal microbalance with dissipation (QCM-D), while samples for cell culture were prepared on PS Petri dishes. First, thin PS films were spin-coated on silicon substrates and gold sensors by using a spin coater (WS-650-23; Laurell Technologies Corporation; North Wales, PA, USA). A drop of a PS solution (1 wt %) in toluene was applied to the substrates (50 μL for silicon and 30 μL for sensors) at 3000 rpm and spun for 60 s. Subsequently, the substrates were dried in a vacuum at 400 mbar and 60 °C for 2 h. Hydrogels on PS-coated silicon and gold substrates were prepared by spin-coating 50 μL of the copolymer solution (PHPA-*stat*-ABP: 0.5 wt % for ∼15 nm coatings; 1.5 wt % for ∼50 nm coatings or PNIPAm-*stat*-ABP: 0.5 wt % for ∼15 nm coatings, 2 wt % for ∼100 nm coatings) in EtOH. For the preparation of PHPA-*stat*-ABP as well as PNIPAm-*stat*-ABP-based hydrogels on suspension culture dishes (Falcon PS Petri dishes; Ø 3.5 cm), the dishes were similarly spin-coated at 3000 rpm for 60 s by applying 100 μL of the 0.5 wt % polymer solution in EtOH. Alginate coatings on silicon wafers were prepared by spin coating 50 μL of a 1 wt % sodium alginate solution at 3000 rpm for 120 s and cross-linking *in situ* by dropping a 3 wt % CaCl_2_ solution after 15 s of spinning. The alginate-coated silicon wafers were then placed in Milli-Q water on a shaking plate for 3 h, rinsed with Milli-Q water, and dried at 80 °C.

### UV-Cross-Linking of Spin-Coated BP-Containing Hydrogel Precursors

Covalent cross-linking and simultaneous substrate immobilization of the spin-coated BP-containing polymers (PHPA-*stat*-ABP and PNIPAm-*stat*-ABP) on PS-coated silicon and gold substrates as well as Petri dishes was achieved through UV-light irradiation using a UV-KUB 2 from Kloè (Montpellier, France) with a wavelength of 365 nm and an intensity of 25 mW cm^–2^ (100%) for 320 s. The hydrogel-coated substrates were extracted in EtOH for 18 h, subsequently rinsed with Milli-Q water, and used for further experiments after gentle drying under a stream of N_2_.

### Self-Assembly of Block Copolymers on Hydrogel Coatings

PGE-*block-*BP brush coatings on surface-bound PHPA hydrogels were prepared statically by incubating the hydrogel-coated substrates in 2 mL of the PGE-*block*-BP copolymer solution (250, 125, 62.5, 31.25 μg mL^–1^ for silicon wafers, 250 μg mL^–1^ for Petri dishes, 300 μg mL^–1^ for gold substrates) in aq. EtOH (H_2_O:EtOH 48:52 v/v %) for 1 h in the absence of light. After the supernatant solution was gently discarded from the hydrogel samples, the surfaces were carefully dried under a stream of N_2_ and subsequently irradiated with UV light (UV-KUB 2; 365 nm; 25 mW cm^–2^) for 320 s. The so-prepared bilayer structures were extracted in EtOH until the dry layer thickness measured by spectroscopic ellipsometry (SE) remained constant (∼5–10 h). Analogous brush coatings on surface-bound PNIPAm and alginate hydrogels were prepared with PGE-*block*-BP copolymer solutions of 250 μg mL^–1^ and 1 wt %, respectively. More detailed procedures are provided in the Supporting Information.

### Investigation of Polymer Self-Assembly and Protein Adsorption via QCM-D

For real-time polymer adsorption experiments, preformed PHPA-*stat*-ABP hydrogels on PS-coated QCM-D gold sensors showing a dry layer thickness of 13.6 ± 0.2 nm via SE (*n* = 3) were used and equilibrated in aq. EtOH in the QCM-D flow chamber at 20 °C. Then, dilute solutions (250 μg mL^–1^) of PGE (*M*_n_ = 26 kDa, *Đ* = 1.08) lacking the BP-based anchor block and a corresponding PGE-*block*-BP copolymer in aq. EtOH were passed over the hydrogel-coated sensors under dynamic conditions (0.1 mL min^–1^) for 10 min, followed by a switch to aq. EtOH for ∼20 min to remove nonadsorbed polymers until the frequency shift Δ*f* of the third overtone reached a plateau. For protein adsorption measurements on the hydrogel- and bilayer-coated sensors, DMEM cell culture medium supplemented with 10% FBS was used as a protein-containing medium. The solution was injected into the chamber after establishing a stable baseline with prewarmed PBS buffer at either 20 or 37 °C under a constant flow of 0.1 mL min^–1^. After 20 min of protein-containing medium flow, the system was rinsed with PBS to remove loosely adsorbed proteins until the frequency shift reached a stable plateau again. Detailed procedures are provided in the Supporting Information.

### Cell Adhesion and Proliferation Studies

Human dermal fibroblasts (HDFs) were tested negatively for mycoplasma on a monthly basis and used in passages 3–7 for the experiments. Polymer-coated Petri dishes for cell culture were disinfected with 70% EtOH for 10 min and subsequently washed twice with cold DPBS. Dishes that underwent preincubation with cell culture medium were treated with 2 mL of DMEM+ at 37 °C and 5% CO_2_ for 30 min. Preincubated and nonpreincubated coated dishes and TCPS controls were seeded with HDFs (3.5 × 10^4^ cells cm^–2^) and cultured at 37 °C and 5% CO_2_ for the indicated time with frequent media changes every 2–3 days. Cells were observed via phase contrast microscopy after 4, 24, 48, and 72 h. More detailed procedures are provided in the Supporting Information.

## Preparation and Surface Modification of PHPA-Based Bulk Gels

### Synthesis of PHPA-*stat*-ABP-Based Bulk Gels and Surface Modification with PGE-Brushes

PHPA-*stat*-ABP-based bulk hydrogels were prepared from a solution of PHPA-*stat*-ABP (500 mg mL^–1^) in EtOH. 250 μL each were placed into the compartments (7 mm × 7 mm × 4 mm) of a self-casted silicone mask sandwiched between two microscope glass coverslips. The samples were irradiated with UV light (UV-KUB 2; 365 nm; 25 mW cm^–2^) for a total of 26 min. Irradiation was accomplished from the upper and lower sides (13 min each) to avoid a cross-linking gradient throughout the samples. PGE-*block-*BP brush coatings on PHPA-*stat*-ABP bulk gels were prepared by incubating the gel substrates in 3 mL of the PGE-*block*-BP copolymer solution (250 μg mL^–1^) for 1 h in the absence of light. After incubation, the samples were gently dried under a stream of N_2_ and directly irradiated with UV light (UV-KUB 2; 365 nm; 25 mW cm^–2^) for 320 s. The detailed procedures are provided in the Supporting Information.

### Synthesis of PHPA-MBAA Bulk Gels and Surface Modification with PGE-Brushes

For the free radical cross-linking polymerization of PHPA-MBAA bulk gels, the cross-linker MBAA was placed in a glass vial together with 1 mol % AIBN and dissolved in MeOH (70 wt %). Then, HPA was added to the reaction mixture. The reaction was initiated by placing the vial in a preheated oil bath at 50 °C for 24 h. PGE-*block-*BP brush coatings on PHPA-MBAA gels were prepared by incubating the bulk gel substrates in 3 mL of the block copolymer solution (300 μg mL^–1^) for 1.5 h in the absence of light. After incubation, the samples were gently dried under a stream of N_2_ and directly irradiated with UV light (UV-KUB 2; 365 nm; 25 mW cm^–2^) for 5 min each from the top and bottom sides by flipping the samples upside down after 5 min. The detailed procedures are provided in the Supporting Information.

### PHPA-MBAA Bulk Gel Swelling

The swelling ratio of PHPA-MBAA_3%_ bulk gels was evaluated in Milli-Q water and in the H_2_O:EtOH mixture (48:52 v/v %) at RT (20 °C). The solvent-equilibrated gels were weighed after removing excess solvent. The swelling ratio SR was calculated according to eq 1,
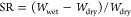
1where *W*_wet_ and *W*_dry_ correspond to the mass of the equilibrated wet and dry (48 h at 60 °C) gels, respectively.

### Diffusive Adsorption Capacity of Bulk Gels for Lucifer Yellow

The diffusive adsorption capacity of pristine and brushed-up PHPA-MBAA bulk gels was evaluated by using the fluorescent dye Lucifer Yellow (LY). The extracted samples were incubated for 18 h in 300 μL of an aqueous LY solution (100 μg mL^–1^) at 4 °C under protection from light. A calibration curve was prepared by measuring the fluorescence intensity of aqueous LY solutions at concentrations of 3.12, 6.25, 12.5, 25, 50, 75, and 100 μg mL^–1^. After the gel samples were removed from the dye solution, aliquots of 100 μL were transferred to a black 96-well plate in duplicates. Quantitative analysis of the collected aliquots was performed with a multimode TECAN Spark plate reader (Männedorf, Switzerland) in fluorescence mode with the appropriate excitation and emission wavelength. Gains were manually set to 36 to compensate for the broad concentration range. The adsorption capacity (*q*_e_) for LY in μg dye/mg adsorbent was calculated using eq 2, where *C*_0_ (μg mL^–1^) and *C*_e_ (μg mL^–1^) are the initial and equilibrium concentrations of the LY solution before and after 18 h of absorption, respectively, *V* (mL) is the volume of the LY dye solution, and *m* (mg) is the mass of the dried gel sample (dried at 60 °C for 48 h).
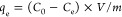
2

## Characterization

Supporting Information provides details of nuclear magnetic resonance (NMR) spectroscopy, gel permeation chromatography (GPC), and microscopy analysis.

### Spectroscopic Ellipsometry (SE)

For the characterization of PS films, the dry layer thickness and the refractive index were measured by SE at an incident angle of 70° and wavelengths from 370 to 1070 nm with a SENpro spectroscopic ellipsometer from SENTECH Instruments GmbH (Berlin, Germany) and calculated as an average value of five different spots on the surface and further used as fixed values for the subsequent modeling of the hydrogel layer. The dry thickness of PHPA-*stat*-ABP, as well as PNIPAm-*stat*-ABP hydrogel layers, was determined similarly as an average of five different spots on the surface by fitting a model consisting of a silicon dioxide layer, a PS layer with fixed parameters, and a Cauchy layer – the layer to be determined – with a fixed refractive index *n* = 1.45 (PHPA-*stat*-ABP) and *n* = 1.48 (PNIPAm-*stat*-ABP) and air as the surrounding medium. The dry thickness of brushed-up hydrogels was determined by adding an additional Cauchy layer with a fixed refractive index of *n* = 1.45 on top of the described model above and air as the surrounding medium.

### Water Contact Angle

The wettability of the coatings was determined by static contact angle (CA) measurements with an OCA contact angle system from DataPhysics Instruments GmbH (Filderstadt, Germany) and fitted with the software package SCA202 (ver. 3.12.11) using the sessile drop configuration. CAs of the immobilized hydrogels were determined after extraction of non-cross-linked chains at 20 °C (PHPA-*stat*-ABP and PNIPAm-*stat*-ABP). A drop of Milli-Q water (2 μL) was placed onto the surface, and the CAs were determined right after deposition with the Young–Laplace model. For each substrate, CAs were measured on five different spots to test for sample homogeneity and on at least four independent substrates (*n* = 4) to test for reproducibility. More detailed procedures can be found in the Supporting Information.

### X-Ray Photoelectron Spectroscopy (XPS)

XPS analysis was performed with an EnviroESCA spectrometer (SPECS Surface Nano Analysis GmbH, Berlin, Germany), equipped with a monochromatic Al Kα X-ray source (excitation energy = 1486.71 eV) and a PHOIBOS 150 electron energy analyzer set to fixed analyzer transmission (FAT) mode. All spectra were acquired under near-ambient pressure (NAP) conditions, applying a water atmosphere of 3 mbar for surface charge neutralization. The spectra were measured in normal emission, and a source-to-sample angle of 55° was used. The binding energy scale of the instrument was calibrated, following a technical procedure provided by SPECS Surface Nano Analysis GmbH (calibration was performed according to ISO 15472). Survey spectra were acquired with a pass energy of 100 electronvolts (eV), and the highly resolved XP spectra were acquired with a pass energy of 50 eV. Raw data were fitted using UNIFIT 2020 data processing software. For fitting, a Shirley background and a Lorentzian–Gaussian sum function were used. If not denoted otherwise, the L-G mixing component was set to 0.30 for all carbon peaks and 0.40 for all heteroatom peaks. If not denoted otherwise, all binding energies were referenced to the binding energy of sp^3^-hybridized C–C bond component at 285 eV.

### Time-of-Flight Secondary Ion Mass Spectrometry (ToF-SIMS)

ToF-SIMS analysis was conducted on a TOF-SIMS M VI instrument manufactured by IONTOF GmbH (Münster, Germany). The samples were measured at RT. The investigations were performed in collimated burst alignment mode using a 25 kV Bi_3_^+^ beam in positive polarity. A region of interest (ROI) of 100 × 100 (μm) was rastered in the sawtooth mode with 256 × 256 pixels and one shot per pixel. An argon secondary ion gun in the 5 keV mode with Ar_1100_ clusters was used for 3D measurements. The crater size was set to 500 × 500 μm. 3D measurements were performed in the noninterlaced mode. The spectra were calibrated to typical organic fragments (CH_2_^+^ (14.02), C_2_H_4_^+^ (28.03), C_3_H_6_^+^ (42.05), and C_4_H_8_^+^ (56.06)). The acquired SIMS data was analyzed using Surface Lab 7.3 software (IONTOF GmbH, Münster, Germany). For 3D rendering, either no binning (for the renders of total ion counts) or a 4 times binning (for all fragments) was used.

## Results and Discussion

**Figure 1:**
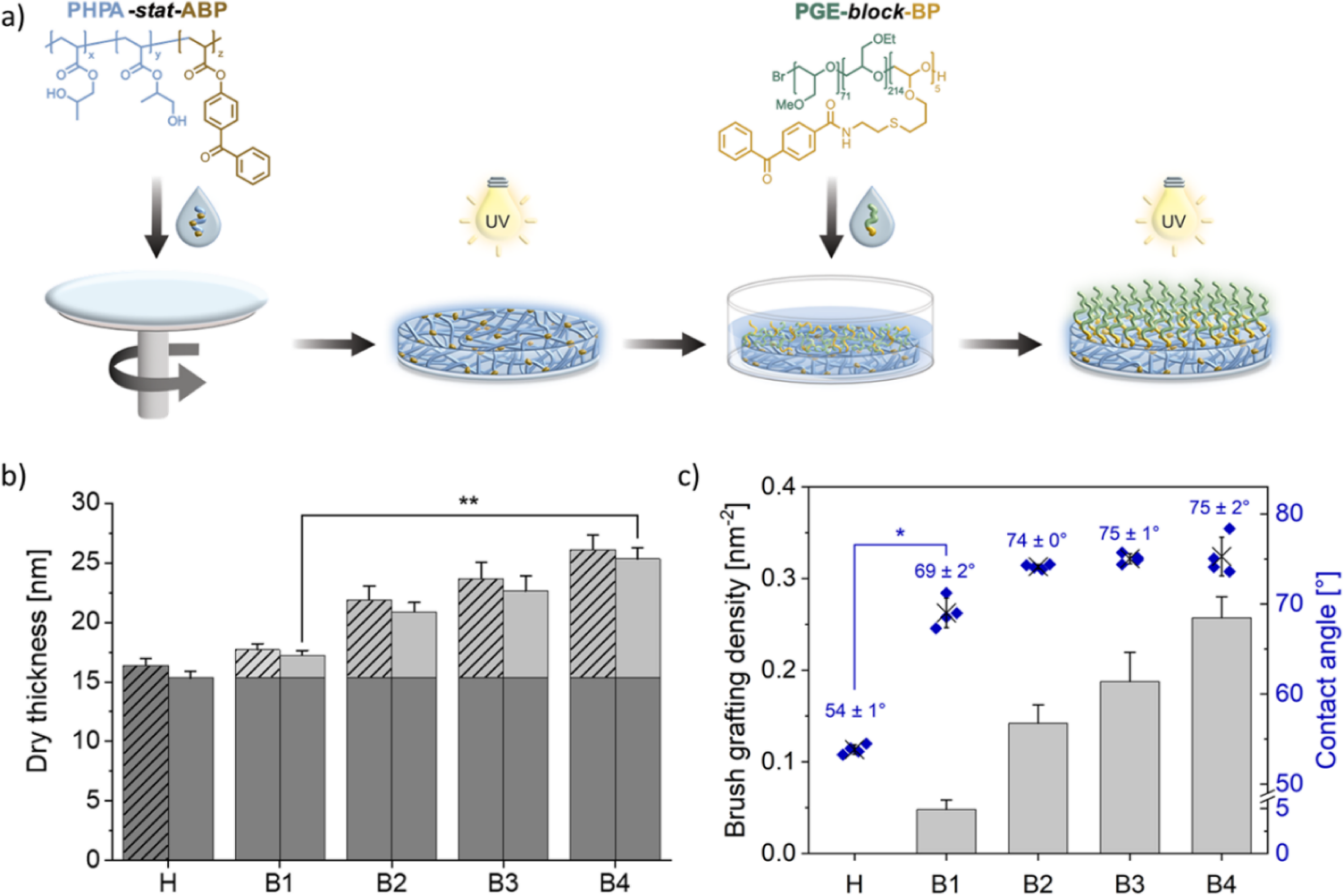
(a) Schematic representation of the “brushing-up” process on spin-coated and UV-cross-linked, surface-immobilized PHPA-*stat*-ABP thin-film gels via a “grafting-to” approach using the self-assembly potential of PGE-*block*-BP copolymers with subsequent UV light-induced (λ = 365 nm, 320 s) covalent immobilization on the hydrogel substrate. (b) Dry layer thickness after UV-immobilization (patterned) and extraction in ethanol (solid) of surface-bound PHPA-based hydrogels (**H**, dark gray) and brushed-up gels (**B**, light gray) produced via self-assembly of PGE-*block*-BP at concentrations of 31.25 (**B1**), 62.5 (**B2**), 125 (**B3**), and 250 (**B4**) μg mL^–1^ at RT (20 °C) for 1 h determined on PS-coated silicon wafer substrates. (c) Corresponding brush GDs (left-y-axis, bars) on 15 nm hydrogel films after extraction in EtOH and their representative static water CA (right y-axis, diamonds). Error bars indicate the standard deviation (SD). (*n* ≥ 4) Statistical significance was tested with a nonparametric Kruskal–Wallis ANOVA test. (*, *p* < 0.05; **, *p* < 0.005).

To test our hypothesis that amide-group-containing BP anchor blocks can drive the self-assembly of block copolymers on swollen and soft hydrogel substrates, we initially focused on surface-bound hydrogels to facilitate convenient analysis via surface-analytical tools. We first selected photochemically cross-linked poly(hydroxypropyl acrylate) (PHPA)-based gels as a fully synthetic model substrate. Therefore, the synthesis of a high molecular weight statistical copolymer PHPA-*stat*-ABP containing 2 mol % of the photo-cross-linkable comonomer 4-acryloyloxybenzophenone (4-ABP) was established (Scheme S2). The great benefit of such BP-containing statistical copolymers is the possibility to cross-link their spin-coated films through UV light-initiated C,H-insertion, which simultaneously immobilizes the forming hydrogel on the plastic support as illustrated in Figure 1a.^26^ For the self-assembly on the hydrogel coatings, PGE-*block*-BP copolymers (Figure 1a)^24^ with an average molecular weight of *M*_n_ ∼ 30 kDa and an average of 5 BP units per anchor block were employed. These block copolymers hold the potential for UV light-initiated covalent surface immobilization after brush formation (Figure 1a), as previously demonstrated on various hard plastic substrates.^25,34−36^

To allow for distinct surface and functional analysis, brushed-up gels were prepared on PS-coated silicon wafers and QCM-D sensors as well as PS Petri dishes. The cell-repellent and antifouling behavior of thin films composed of PHPA hydrogels makes them ideal substrates to prove successful “brushing-up” with intrinsically cell-adhesive PGE brushes via their performance in cell culture. Although PHPA exhibits LCST behavior,^37,38^ we herein do not focus on the gel’s thermoresponsive properties but aim to selectively tune its interface. The thermoresponsive properties of the PGE block copolymer with a cloud point temperature (*T*_cp_) of 20 °C^24^ can be further used to demonstrate the functionality of the generated bilayers in cell culture by thermally triggered cell sheet detachment as previously shown with PGE brushes on hard substrates.^23,25,34,36^

### PGE Brushes on PHPA-Based Thin Films

A mandatory requirement to efficiently produce gel coatings via the illustrated approach in Figure 1a is a statistical BP-containing copolymer of high molecular weight (>30 kDa). The highest reported number-average molecular weights (*M*_n_) of PHPA in the literature range between 10 and 15 kDa, regardless of the polymerization method employed.^33,39−42^ Therefore, the conditions of the free radical polymerization were optimized by adjusting the initiator and monomer concentrations and the reaction temperature, as summarized in Table S1. This yielded a high molecular weight PHPA-*stat*-ABP copolymer (*M*_n_ ∼ 50 kDa) with acceptable dispersity (*Đ* ∼ 3) (Table S1), sufficient for efficient gel formation (Figure S4). To fabricate PS-bound hydrogel layers, a 0.5 wt % PHPA-*stat*-ABP solution in ethanol was spin-coated onto PS substrates and subsequently immobilized by short UV irradiation. After extraction of the non-cross-linked polymer chains with ethanol, the resultant hydrogels (**H**) showed a dry thickness of approximately 15 nm, as measured by spectroscopic ellipsometry (SE) on coated silicon wafers (Figure 1b). Static water contact angle (CA) measurements at room temperature (RT) revealed a significant increase in PS surface wettability from ∼90° to 54 ± 1° (*n* = 6) after PHPA-*stat*-ABP immobilization, confirming the intrinsic hydrophilicity of the hydrogel.

To self-assemble a PGE-*block*-BP brush monolayer on top of hydrogel **H**, the substrates were incubated with dilute PGE-*block*-BP solutions of varying concentrations in a selective solvent mixture. As previously reported for such BP-functionalized PGE block copolymers, using aqueous ethanolic solutions (here: 48 v/v % water) as a selective solvent enhances the directionality of the anchor block toward hydrophilic substrates.^25^ To assess the efficiency of the self-assembly and photoimmobilization process, the dry layer thickness of the generated and UV-irradiated bilayer structures (**B1–4**) on 15 nm gels was determined by SE before and after extracting nonattached chains in ethanol (Figure 1b). It is worth mentioning that a fair amount of interchain cross-linking during UV irradiation could occur due to the close proximity of the self-assembled BP anchor units on the gel surface, potentially enhancing the stability of the PGE brush layer. The corresponding brush GDs, as well as water CAs on the pristine hydrogel and bilayers, are shown in Figure 1c. Similarly to the self-assembly of PGE-*block*-BP brushes on TCPS,^25^ the GD on PHPA-*stat*-ABP gels could be tuned via the block copolymer concentration in the range of 0.05–0.26 chains nm^–2^. Considering the high *M*_n_ of the PGE-*block*-BP copolymer and the solvent-swollen dynamic hydrogel substrate, such high GDs are remarkable and significantly higher than, e.g., PNIPAm-based brushes of similar *M*_n_ grafted onto hard substrates via a dopamine priming layer^43^ or other “grafting-to” strategies.^44^ However, when employing a thicker dopamine priming layer impressively high GDs up to 0.48 chains nm^–2^ on hard substrates could be achieved.^45^ Although GDs as high as 1.13 chains nm^–2^ have been reported for “grafting-from” approaches on hard surfaces,^7^ efficient surface-initiated polymerization (SIP) is largely restricted to (meth)acrylate monomers. Surface functionalization by SIP with polymers produced via anionic living polymerization, such as poly(ether)-based polymers, is not easily accomplished on hard substrates and impossible on swollen hydrogels.^46^ Thus, despite lower GDs compared to “grafting-from”, our method successfully achieved covalent immobilization of PGE-based brushes, not yet achieved with SIP techniques.

Control experiments with alternative PGE-*block*-BP copolymers, which did not contain an amide but an ether linkage between the BP unit and the polymer chain,^24^ failed to produce dense brushes on the gel surface (Figure S5), highlighting the importance of amide group-specific interactions between anchor and hydrogel. After ethanolic extraction of the UV-irradiated self-assembled brushes, the layer thickness decreased by only approximately 15% (Figure 1b). Thus, the BP-based anchor block effectively directed the self-assembly process toward the hydrogel surface, as similarly observed on hard TCPS substrates.^25,34^ As type II photoinitiators, BP units facilitated their light-induced C,H-insertion into the hydrogels’ polymer chains located at the gel–brush interface. This is remarkable given the short lifetime and low stability of the formed BP radicals in the presence of excessive solvent molecules.^47^ To confirm that the efficient brushing-up of the PHPA-*stat*-ABP hydrogel occurred in its swollen state and was not assisted by a potential collapse of the gel under the grafting conditions, we assessed its layer thickness in water and aqueous ethanolic solution (48:52 v/v) using SE. Expectedly, the gel was dehydrated at 20 °C due to its thermoresponsiveness in water but remained solvated in the selective solvent (Figure S6).

Brushing-up the hydrogel surface decreased its wettability with increasing GD, approaching a CA of approximately 75° (**B2–4**) when the dry brush thickness exceeded 6 nm (Figure 1c). At lower GDs (**B1**), CAs around 70° suggested partial coverage of the hydrogel surface, which was further supported by polymer theory-based estimations of the brush conformation (Figure S7).^25,48^ Considering the degree of the chain overlap, 2*R*_f_*l*^–1^, with the PGE block copolymer’s Flory radius *R*_f_ and its anchor distance *l* within the brush layer suggests full hydrogel coverage at polymer concentrations of 62.5 μg/mL or higher. This estimation holds true for PGE brushes on PHPA hydrogels above and below the *T*_cp_ of PGE as assessed with the respective *R*_f_ values in a bad (37 °C) or theta (20 °C) solvent (further details can be found in the Supporting Information).

**Figure 2:**
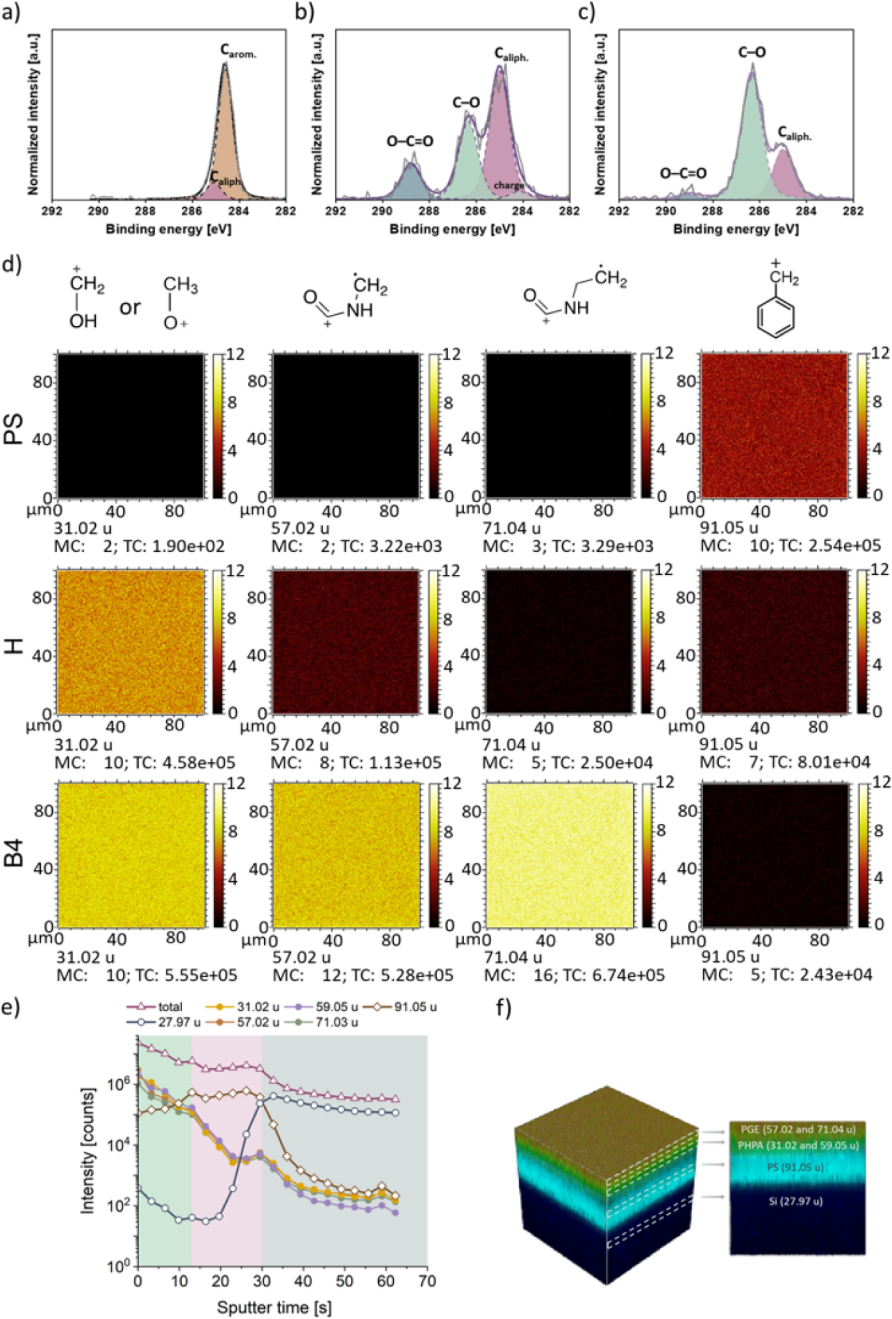
(a) Highly resolved C 1s XP spectra (gray line) of the basal PS substrate, (b) PHPA-*stat*-ABP gel **H** (15 nm) immobilized on PS, and the (c) final bilayer **B4** immobilized on PS. Fitted peak components and the sum curve of all fitted peak components are illustrated by the purple dashed and solid lines, respectively. (d) ToF-SIMS analysis of the basal PS-substrate, the PHPA-*stat*-ABP gel (15 nm) (**H**) immobilized on PS, and the final PS-immobilized bilayer **B4**. ToF-SIMS images of characteristic fragments of the PS-coated wafer were obtained after each modification step with the maximum counts per pixel (MC) and total counts (TC). Scalebar indicates the ion count per pixel. (e) Depth profile of the number of the positively charged mass fragments detected during time-dependent sputtering of the bilayer **B4** with Ar_1100_-clusters. (f) 3D rendered image from the positively charged ion intensities of the characteristic layer fragments in the brushed-up structure **B4** on PS from ToF-SIMS 3D measurement.

To confirm the successful bilayer formation and the spatial restriction of PGE-*block*-BP brush functionalization to the surface of the PHPA-based hydrogels, XPS characterization was performed on PS-coated silicon wafers after each functionalization step (Figure 2).^5^ The highly resolved C 1s XP spectrum of the basal PS substrate clearly showed the expected aromatic (C_arom._ 88%) and aliphatic (C_aliph._ 12%) components at 284.6 and 285.0 eV, respectively (Figure 2a and Table S2). After immobilizing the PHPA-*stat*-ABP gel (**H**, ∼ 15 nm) on the PS surface, besides the C_aliph._ (52%) component, additional peak components appeared at 286.3 eV (31%) and 288.8 eV (14%) corresponding to C–O and O–C=O bonds, respectively (Figure 2b and Table S2). Further functionalization with the PGE brushes markedly altered the peak ratio in the C 1s spectrum of the final bilayers **B4** (Figure 2c and Table S2). The apparent decrease of the characteristic acrylic ester signals (O–C=O components) derived from the basal gel after PGE grafting indicated a brush thickness of ≥10 nm, which was in agreement with the dry layer thickness determined via SE (Figure 1b).^49^ Expectedly, increasing the basal gel thickness from 10 to 50 nm did not alter the peak ratios (Figure S8a and Table S2). A substantial decrease of the acrylic O–C=O component at 289.0 eV and an increase of the polyether-derived C–O component at 286.3 eV after PGE-*block*-BP immobilization on the thin as well as thicker PHPA-based gels confirmed the formation of brushed-up structures, irrespective of the gel thickness (Figure S8b and Table S2). Further analysis of the bilayer sample **B2** with lower brush GD yielded similar C 1s spectra, proving successful surface grafting on the gels (Figure S8c and Table S2).

ToF-SIMS analysis provided detailed information on the chemical composition and spatial distribution of the fabricated bilayers.^50^ PS-coated Si wafers were investigated after each functionalization step by using different measurement modes, including 2D and 3D imaging (Figure 2d–f). The SIMS spectrum of the basal PS substrate showed an intense peak at 91.05 u, originating from the C_7_H_7_^+^ benzyl fragment (Figure 2d and S9).^51^ After immobilization of the PHPA-*stat*-ABP hydrogel layer **H**, the intensity of the benzyl fragment markedly decreased, while peaks at 31.02 u (HOCH_2_^+^) and 59.05 u (C_3_H_7_O^+^, Figure S9), indicative of the hydroxy-functional polymer gel residues, appeared. Subsequent surface functionalization with PGE brushes further decreased the C_7_H_7_^+^ intensity and increased the peak at 31.02 u due to the methoxy CH_3_O^+^ fragment of the GME comonomer. Additionally, new peaks at 57.02 u (C_2_H_3_NO^•^^+^) and 71.04 u (C_3_H_5_NO^•^^+^), characteristic of the amide moieties in the PGE-*block*-BP anchor block, emerged (Figure 2d).

The uniform distribution of the polymer fragments across the layers indicated a laterally homogeneous polymer coating. Argon cluster sputtering for depth profile measurements provided additional interfacial information.^52^ For bilayer **B4**, the characteristic fragments of PHPA and PGE layers gradually decreased over the first 13 s of sputtering, thus exposing the basal PS layer (benzyl^+^ fragment arose), which was completely removed after 30 s of sputtering (Figure 2e). The rise of Si^+^ fragments from the underlying wafer became apparent between 20 and 32 s of sputtering. Although the plain 3D rendering does not precisely reflect the actual layer thicknesses, the 3D-rendered image of the ion counts characteristic for the fragments in the different layers (Figure 2d) confirmed the formation of the organic bilayer structure on PS-coated wafers. The sequential stacking of layers on the wafer was further supported by the declining intensities of the benzyl^+^ and Si^+^ fragments with each functionalization step (Figure S10).

**Figure 3:**
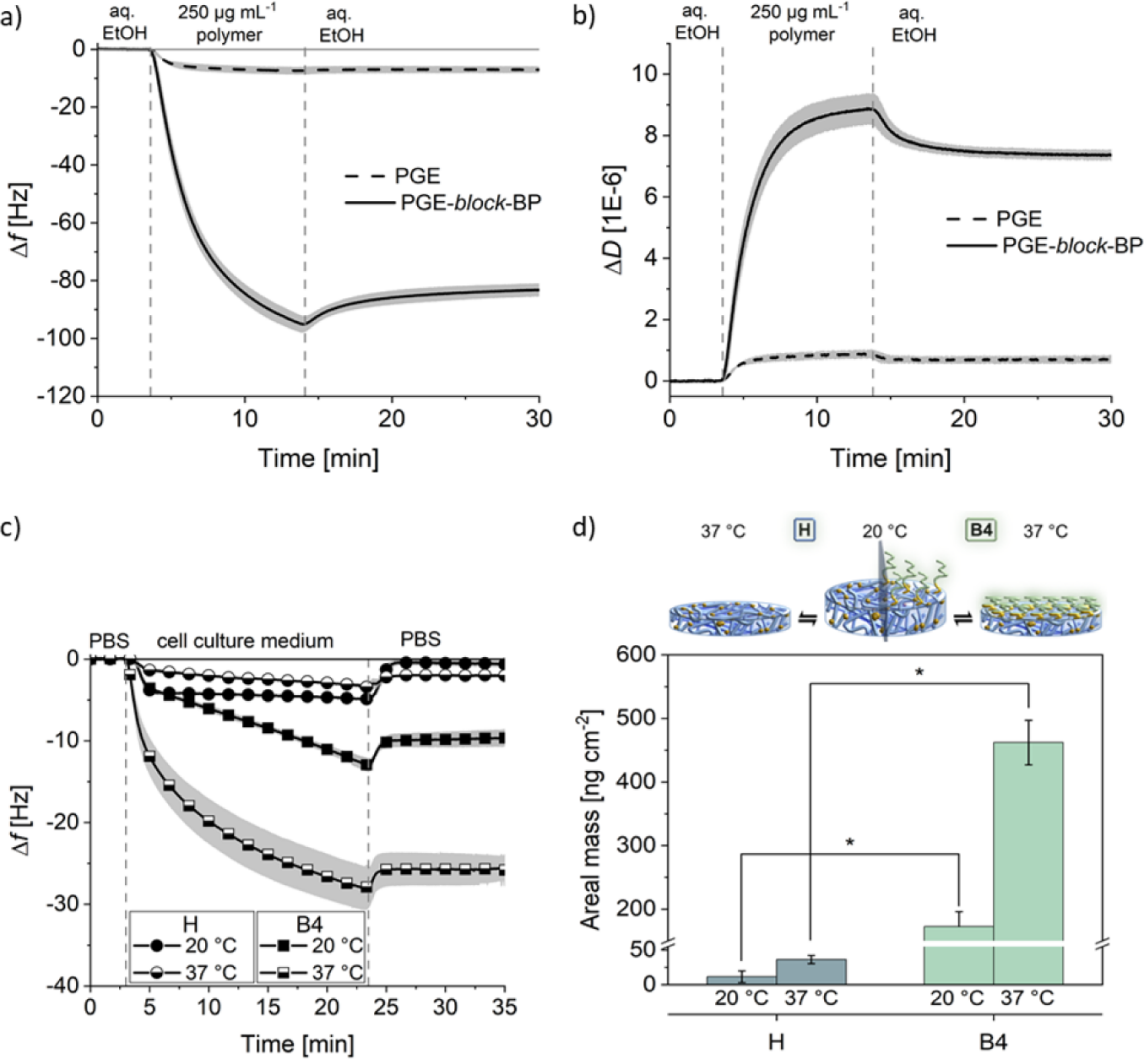
QCM-D adsorption experiments on the PHPA-*stat*-ABP hydrogels **H** and their brushed-up counterparts **B4** immobilized on PS-coated sensor chips. Vertical dashed lines indicate medium changes. Representative (a) frequency shift (Δ*f*) and (b) dissipation shift (Δ*D*) curves for the adsorption of PGE lacking the BP-based anchor block (dashed line) and PGE-*block*-BP (solid line) from aq. EtOH (48:52 v/v %) solutions (250 μg mL^–1^) on the pristine gels **H**. (c) Dynamic protein absorption from cell culture medium supplemented with 10% FBS on hydrogels **H** (circles) as well as on the brushed-up gels **B4** (squares) at 20 °C (filled) and 37 °C (half-filled) monitored by recording Δ*f* for the 3rd overtone of the sensor. Data are shown as average ± SD (gray shadow along curves). (d) Schematic illustration depicting the physical changes occurring in the polymer brush layer and the underlying hydrogel with temperature changes and residual protein adsorbed on gels **H** (blue) and bilayer **B4** (green) at 20 and 37 °C calculated with the Sauerbrey model at *t* = 35 min. Data are plotted as average ± SD, (*n* = 3). Statistical significance was tested with a nonparametric Kruskal–Wallis ANOVA test (*, *p* < 0.05).

To further confirm that the interaction between the self-assembling block copolymers and hydrogels is primarily due to the anchor block rather than the PGE backbone itself, the adsorption of PGE copolymers was studied via QCM-D in the presence and absence of the anchor block. Comparing representative frequency (Δ*f*) and dissipation (Δ*D*) shifts during and after exposure of PHPA-based hydrogels to PGE and PGE-*block*-BP in aqueous ethanol under flow revealed a substantially stronger interaction with the BP-containing copolymer (Figure 3a,b), consistent with analogous experiments on hard PET or TCPS surfaces.^24^

In a first functional assessment, the temperature-dependent interaction of biological fluids with **B4** bilayers (GD ∼ 0.21 chains nm^–2^) and their pristine counterparts **H** was evaluated. Therefore, fetal bovine serum (FBS)-supplemented cell culture medium was passed over the coated sensors at 0.1 mL min^–1^, recording Δ*f* and Δ*D* at 20 and 37 °C (Figures 3 and S11). While the proteins from the medium barely interacted with the PHPA-*stat*-ABP gel, Δ*f-*curves markedly increased during 20 min of bilayer exposure to cell culture medium, revealing stronger protein interactions at both 20 and 37 °C (Figure 3c). After rinsing the surfaces with PBS, protein retention was negligible on the pristine PHPA gel but substantial on the bilayers, especially at 37 °C. The drastic increase in protein adsorption on the bilayers between 20 and 37 °C (Figure 3d) indicates the thermal switchability of the PGE brush coating, similarly observed on hard substrates.^25,34,53^

**Figure 4:**
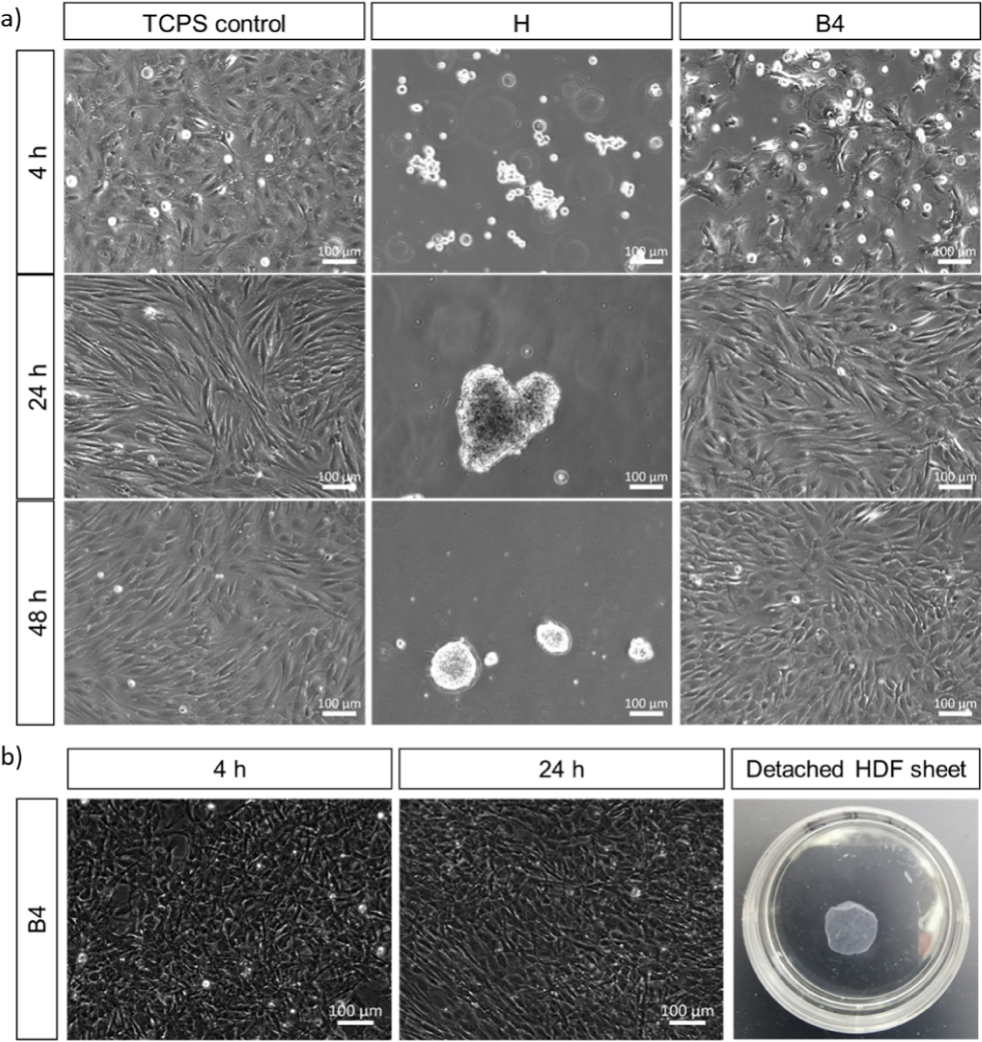
(a) Representative phase contrast images of HDFs 4, 24, and 48 h after the seeding on a TCPS control, PHPA-*stat*-ABP hydrogel **H**, and bilayer **B4** coated substrates. Dishes were preincubated in cell culture medium supplemented with 10% FBS for 30 min at 37 °C before seeding. Seeding density: 3.5 × 10^4^ cells cm^–2^. (b) Representative phase contrast images of HDFs 4 and 24 h after seeding on **B4** and respective macroscopic photograph of a spontaneously detached HDF sheet at RT in the cell culture dish. Seeding density: 1.35 × 10^5^ cells cm^–2^ (*n* = 3).

The functional impact of the differential protein adsorbing properties became more evident when both surfaces were employed as substrates for the culture of HDFs. Within 48 h, HDFs adhered and grew to confluency on **B4** after pretreatment with cell culture medium for 30 min at 37 °C before cell seeding, similar to the TCPS control. In strong contrast, cells displayed poor adhesion on pretreated PHPA-*stat*-ABP gels, forming clusters and spheroids within 24 h (Figure 4). Notably, HDFs cultured for 48 h on nonpretreated **B4** showed similar confluency (∼90%) as pretreated bilayers (Figure S12), confirming the intrinsic cell adhesive properties of PGE-based functionalized surfaces and the antifouling properties of PHPA gels. Additional fluorescent imaging of live/dead stained HDFs after 48 h culture on nonpretreated **B4** revealed no evidence of acute cytotoxicity of the coating (Figure S12). HDFs cultured on lower GD bilayers (**B1**–**B3)** did not reach confluency even after 72 h (Figure S13), but their proliferation strongly correlated with the PGE GD, confirming improved cell adhesive properties compared with the cell-repellent PHPA hydrogel.

We further investigated the thermal switchability of the PGE brush layer on the gel substrate. Given the outstanding performance of the bilayers **B4** in cell culture, we set the thermally induced, nonenzymatic detachment of confluent HDF sheets as a simple functional read-out. Intact HDF sheets generated within 24 h of culture at 37 °C spontaneously detached when the dishes were placed on the bench at RT (Figure 4b). As similarly observed with thermoresponsive hard substrates, cell sheets started to detach on the outer area of the soft substrate (Video S1) and progressed evenly across the dish (Video S2), completing detachment within 6 ± 4 min (*n* = 6) compared to detachment times of around 30 min on TCPS.^25^ Notably, HDF sheets detached from **B4** bilayers solely by reducing the temperature, without additional triggers such as switching from cell culture medium to PBS, which can cause undesired detachment of immature sheets during microscopic analysis. We attribute the observed accelerated detachment from brushed-up bilayers compared to simple brushes on more hydrophobic hard substrates to enhanced polymer rehydration, facilitated by the hydrophilic gel substrate.

### PGE Brushes on PNIPAm-Based Thin Films

To showcase the versatility of our “grafting-to” strategy beyond PHPA-based substrates, we extended the approach to PNIPAm-based hydrogel coatings made from PNIPAm-*stat*-ABP containing 2 mol % of ABP in an analogous fashion. Details of the PNIPAm-*stat*-ABP synthesis (Scheme S3 and Figure S3) and the preparation of the hydrogel coating can be found in the Supporting Information. Surface-bound gels on PS substrates with a dry layer thickness of ∼17 nm were brushed-up (5 nm) with PGE-*block*-BP in aqueous ethanol (48 v/v % H_2_O) (Figure S14). Similar to PHPA-based bilayers, preincubated PNIPAm-based bilayers comprising PGE brushes facilitated the adhesion and proliferation of HDFs to confluency within 72 h of culture, contrasting with unfunctionalized PNIPAm gels that were cell-repellent (Figure S15).

Swelling studies revealed the ability of PNIPAm-*stat*-ABP coatings to undergo significant swelling in water (2.4 ± 0.3) and aqueous ethanolic solution (1.1 ± 0.1) (*n* = 3, Figure S16b), substantiating the feasibility of the self-assembly process on swollen coatings.

### PGE Brushes on Alginate-Based Thin Films

To further demonstrate that our grafting-to strategy is not limited to covalently cross-linked, BP-containing gel coatings, we applied the methodology to physically cross-linked alginate coatings. Hence, alginate coatings (147.0 ± 4.2 nm) were prepared on silicon wafers by spin coating of a sodium alginate solution (1 wt %) and cross-linked *in situ* with CaCl_2_ (3 wt %) while spinning. By following the same procedure as for PHPA- and PNIPAm-based gels, 8.7 ± 0.1 nm PGE-*block*-BP immobilized on the alginate coatings after ethanol extraction could be detected via SE (*n* = 2, data not shown). XPS analysis of the plain alginate coatings revealed the expected C–C, C–O, and O–C=O contributions at 285.0, 286.6, and 288.3 eV (Figure S17a), respectively, in agreement with the literature.^54,55^ Besides the apparent increase of the C–O component in the C 1s spectra, PGE-*block*-BP functionalization markedly decreased the basal alginate-derived O–C=O components, confirming the presence of a relatively thick PGE brush coating (Figure S17b). As UV irradiation is essential for the covalent immobilization of the BP-containing PGE block copolymer, a UV-irradiated, nonfunctionalized alginate coating was used as a negative control to assess potential spectral changes due to irradiation. Although spectral changes were detected upon UV irradiation, suggesting light-induced surface oxidation of the alginate gels, the intensity of the O–C=O component at 288.3 eV was not markedly affected (Figure S17c).

### PGE Brushes on PHPA-Based Bulk Gels

**Figure 5:**
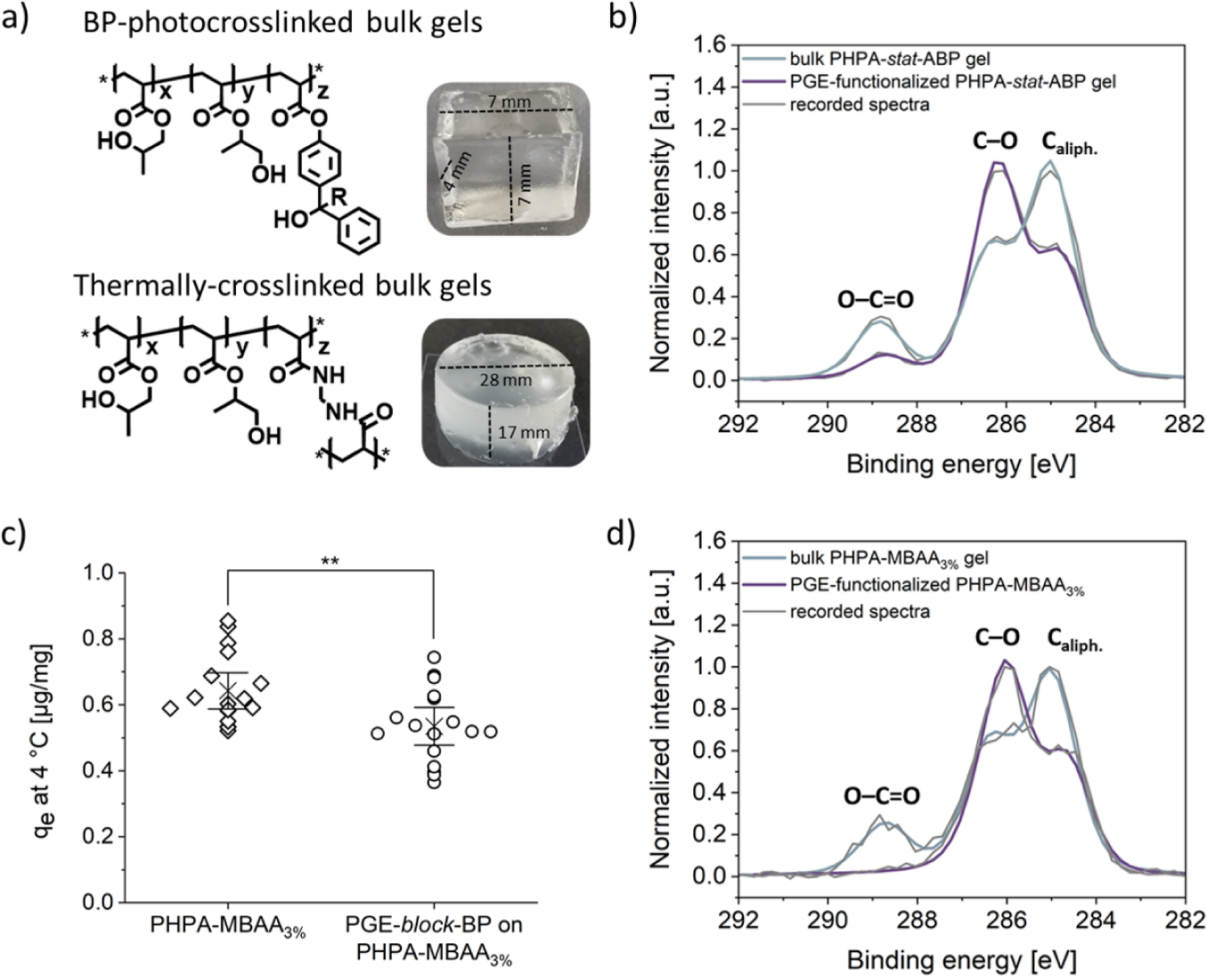
Chemical structure and macroscopic photographs of (a) photo-cross-linked PHPA-*stat*-ABP and thermally cross-linked PHPA-MBAA_3%_ (containing 3% of *N*,*N*′-methylene bis(acrylamide) (MBAA) as the comonomer for efficient cross-linking) bulk gels and (b,d) their respective background subtracted and normalized highly resolved C 1s XP spectra including sum curves of nonfunctionalized (blue) and PGE-brush functionalized (purple) bulk gels. (c) Equilibrium adsorption capacity *q*_e_ of brushed-up (circles) and pristine (diamonds) PHPA-MBAA bulk gels for lucifer yellow (LY) after 18 h of incubation at 4 °C. Data are plotted as independent values along with the 95% confidence interval (whiskers) and mean values (cross). Statistical significance was tested with a paired sample *t* test. (**, *p* < 0.01).

As a final proof-of-concept, we challenged our grafting-to strategy and applied it to bulk hydrogels to validate its efficacy. Both BP- and bis(acrylamide) cross-linked bulk PHPA gels (Figure S18) were used for the interfacial PGE-functionalization, with the latter ensuring that the self-assembly process is not limited to BP-containing hydrogel substrates. Detailed procedures for bulk gel preparation (Scheme S4) and functionalization can be found in the Supporting Information. The compositional changes at the bulk gel interfaces upon PGE-functionalization were investigated via XPS (Figure 5). Similar to PHPA-based hydrogel coatings, the highly resolved C 1s XP spectrum of PHPA-*stat*-ABP-based bulk gels (Figure 5b) showed the characteristic contributions at 285.0 (C_aliph._, 54%), 286.4 (C–O, 31%), and 288.8 eV (O–C=O, 14%). The peak ratio significantly changed after surface functionalization of the photo-cross-linked bulk gel with PGE-*block*-BP. While the relative area of C–O signal increased to 61%, the components arising from C_aliph._ and O–C=O decreased to 33% and 6%, respectively, confirming the presence of a thin (<10 nm) PGE-based brush layer on the surface of PHPA-*stat*-ABP bulk gel as components of the basal gel substrate were still detectable.

Further XPS analysis on the brushed-up and pristine PHPA-*stat*-ABP bulk gels according to the “cutting-edge” method (Figure S19a) confirmatively excluded any diffusion of the PGE block copolymer into the bulk gel matrix. As the spectral profiles of the two freshly cut samples were essentially indistinguishable (Figure S19b), we inferred that the PGE block copolymer did not penetrate into the bulk hydrogel matrix but exclusively modified the bulk gel–water interface. Similarly, the surface of BP-free, bis(acrylamide)-cross-linked PHPA-MBAA bulk gels was successfully functionalized with PGE-*block*-BP, as demonstrated by the highly resolved C 1s XP spectra depicted in Figure 5d. Analogous to the alginate coatings, a UV-irradiated, nonfunctionalized PHPA-MBAA bulk gel served as a negative control (Figure S20) and precluded spectral changes due to irradiation. Notably, the observed differential attenuation of the acrylic ester peak at 288.8 eV in the C 1s spectra (Figure 5b,d) suggests tunability of the brush GD on bulk gels similar to surface-immobilized gels (Figure 1) by adjusting the concentration of the block copolymer solution. Gravimetrical swelling analysis of PHPA-MBAA bulk gels in both water and the selective solvent mixture used for the self-assembly of PGE-*block*-BP revealed a 3-fold increase in the swelling ratio in aqueous ethanol (2.8 ± 0.2%) after 90 min (incubation time for the PGE-functionalization) compared to water (1.3 ± 0.1%) (*n* = 3, Figure S21), indicating that the assembly process occurs on swollen PHPA-MBAA gels.

**Figure 6:**
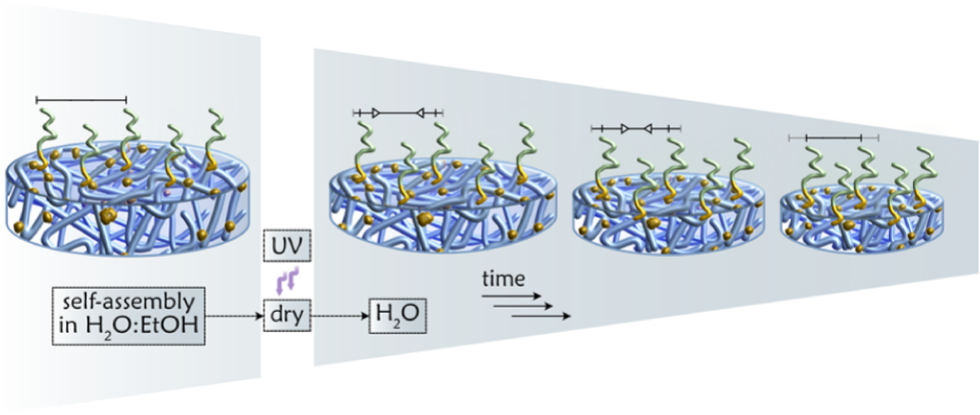
Schematic representation of the GD enhancement via a solvent-induced shrinking process of the gel substrate. This process, applicable to both surface-immobilized and bulk gels, illustrates the structural transition of the gel-based substrates during and after functionalization. Swollen gels in aqueous ethanol undergo shrinking upon exposure to pure water, resulting in a more densely grafted surface. For simplicity, the grafted brushes are illustrated only on the top surface.

To demonstrate the functional impact of bulk gel surface functionalization on the diffusive adsorption capacity of small molecules, we measured the loading capacity of pristine and brushed-up PHPA-MBAA gels for the fluorescent dye LY (Figure 5c). The calculated adsorption capacity *q*_e_ at equilibrium (cf eq 2) revealed a statistically significant reduction in dye uptake upon gel surface functionalization. The additional physical barrier introduced by PGE surface functionalization likely hinders dye diffusion into the bulk of the hydrogel, offering inspiring and exciting possibilities for future applications of PGE-grafted hydrogels. While the equilibrium uptake measurements provide an initial insight into the barrier properties of the PGE-functionalized bulk gels, kinetic dye uptake is currently under investigation. Fibroblast adhesion was additionally evaluated on PGE-*block*-BP functionalized PHPA-MBAA gels, with XPS analysis clearly confirming successful surface modification (see Figure 5d). However, despite a similar brush thickness (approximately 8–10 nm), these brushed-up bulk gels did not support satisfactory cell adhesion, unlike the brushed-up thinner and thus stiffer surface-bound gels. This discrepancy can be attributed to differences in the mechanical properties and surface topography between bulk and surface-immobilized hydrogels. We are currently addressing these points in our ongoing work by optimizing both the grafting process and bulk gel stiffness to enhance cell adhesion and proliferation. Our current efforts to improve the GDs include tuning the solubility of the PGE-*block*-BP during the grafting process (e.g., by performing grafting under cloud-point conditions)^56^ and optimizing the grafting conditions such that the gels remain highly swollen during the self-assembly process but shrink in water postfunctionalization. This shrinkage brings the brush chains closer together, effectively increasing the GD as the gel contracts. This concept is supported by the achieved dry thickness of the PGE brushes on the thin hydrogel coatings. Specifically, after PGE-*block*-BP self-assembly on surface-immobilized PHPA-*stat*-ABP and PNIPAm-*stat*-ABP thin films, we observed a greater PGE brush thickness (∼10 nm vs ∼5 nm) on the highly swollen PHPA-*stat*-ABP (swelling ratio >2) compared to the less swollen PNIPAm-*stat*-ABP (swelling ratio ∼1) film (Figure S16). These results underscore the potential for improving the brush GD through a targeted choice of solvent during the grafting-to approach, as illustrated in Figure 6.

## Conclusion

A versatile and effective brushing-up approach for the surface-restricted modification and functional nanoengineering of soft hydrogels through block copolymer self-assembly and subsequent covalent photoimmobilization was presented. The UV light-induced immobilization process in this grafting-to strategy enables straightforward photopatterning of the hydrogel surfaces in the future. Furthermore, the GD of the resulting brushed-up bilayers can be precisely tuned via the concentration-dependent block copolymer self-assembly from a selective solvent, hence controlling interfacial gel properties as demonstrated for wettability, protein adsorption, and cell behavior. In contrast to established grafting-from approaches, no specific premodification of the hydrogel surface with polymerization initiators is required. Moreover, polymers prepared by anionic living polymerization like PGE, which are challenging to graft by surface-initiated polymerization, can be efficiently grafted onto the surface of hydrogels in their swollen state, containing up to 74% water/ethanol, as demonstrated with PGE block copolymers. Successful PGE brush formation on hydrogel coatings was shown for synthetic PHPA- and PNIPAm-based and natural alginate-based hydrogels. We further extended our interfacial nanoengineering approach to bulk PHPA gels, modulating their bulk properties, as demonstrated by the diffusive adsorption capacity. Notably, the BP-assisted covalent immobilization of the PGE brushes was successfully achieved on the hydrated substrates.

For a functional read-out, the thermoresponsive properties of the PGE brushes were utilized to thermally control protein adsorption on the brushed-up gels, which further allowed HDFs to attach to the substrates and proliferate into confluent monolayers at 37 °C. By lowering the temperature to 24 °C the thermal switchability of the interfacial gel properties was confirmed by rapid spontaneous cell sheet detachment. Thus, the accessible brushed-up gels with a molecularly defined thermoresponsive interface encourage future insightful investigations of the structural parameters governing the reciprocal interactions of the hydrogel substrate and the grafted brush layer. Understanding the mutual, responsive interactions within brushed-up bilayers enables the development of advanced hydrogels with enhanced functionality in terms of friction, permeability, and interaction with biological entities.

## Data Availability

The data that support the findings of this study are available from the corresponding author, [M.W.], upon reasonable request.

## Supporting Information Available

Materials for synthesis; materials for surface modification; materials for cell culture and protein adsorption; methods for synthesis and characterization of 4-ABP; methods for polymers synthesis; methods for surface modification and characterization; preparation and surface modification of PHPA-based bulk gels. Supporting figures: Dry layer thickness of PHPA-based polymers with different molecular weights; dry layer thickness of PGE-*block*-BP brushes with different anchor chemistry immobilized on PHPA-based thin film coatings; solvated layer thickness of PHPA-*stat*-ABP in different solvents; estimated PGE-*block*-BP brush conformation at 20 and 37 °C; highly resolved C 1s spectra for PHPA-*stat*-ABP-based samples; table with XPS fitting parameters, binding energies and peak assignment; ToF-SIMS mass spectra; ToF-SIMS 3D rendering; Δ*D* curves for protein adsorption via QCM-D; microscopic phase contrast images and *L*/*D* staining on B4; microscopic phase contrast images on B1–B3; thickness and wettability of PGE-functionalized and nonfunctionalized PNIPAm-based thin film coatings; microscopic phase contrast images on PGE-functionalized and nonfunctionalized PNIPAm-based thin film coatings; swelling ratio of PHPA-*stat*-ABP and PNIPAm-*stat*-ABP; highly resolved C 1s spectra for alginate-based samples; macroscopic photographs of PHPA-based bulk gels; XPS curves on bulk gels; swelling ratio of PHPA-MBAA in different solvents (PDF)
Outer HDF sheet detachment (AVI)
Inner HDF sheet detachment (AVI)

## Supplementary Material

am4c18632_si_001.pdf

am4c18632_si_002.avi

am4c18632_si_003.avi
