# Supplementary material for: Interfacial Nanoengineering of Hydrogel Surfaces via Block Copolymer Self-Assembly
Source: ACS Appl Mater Interfaces. 2025 Feb 4;17(6):10073–86. doi: 10.1021/acsami.4c18632 (PMC11826506; doi:10.1021/acsami.4c18632)
Supplement: Supplementary file 1 — am4c18632_si_001.pdf [file am4c18632_si_001.pdf]

Supporting information

Interfacial Nanoengineering of Hydrogel Surfaces via Block Copolymer Self-assembly

*Andrea Cosimi,[1,2] Daniel D. Stöbener,[1,2] Philip Nickl,[1,3] Robert Schusterbauer,[1,3] Ievgen S. Donskyi,[1,3] and Marie Weinhart[1,2]**

[1] Institute of Chemistry and Biochemistry – Organic Chemistry, Freie Universität Berlin, Takustraße 3, 14195 Berlin, Germany; E-mail: marie.weinhart@fu-berlin.de

[2] Institute of Physical Chemistry and Electrochemistry, Leibniz Universität Hannover, Callinstraße 3A, 30167 Hannover, Germany; E-mail: marie.weinhart@pci.uni-hannover.de

[3] BAM – Federal Institute for Material Science and Testing, Division of Surface Analytics, and Interfacial Chemistry, Unter den Eichen 44-46, 12205 Berlin, Germany

Table of Contents

1. MATERIALS 3
1.1 Materials for synthesis........ 3
1.2 Materials for surface modification........ 3
1.3 Materials for cell culture and protein adsorption studies........ 5
2. SYNTHESIS AND CHARACTERIZATION OF 4-ACRYLOYLOXYBENZOPHENONE (4-ABP)........ 5
3. SYNTHESIS AND CHARACTERIZATION OF PHPA-*stat*-ABP........ 8
4. SYNTHESIS AND CHARACTERIZATION OF PNIPAm-*stat*-ABP........ 11
5. METHODS FOR SURFACE MODIFICATION AND CHARACTERIZATION........ 13
5.1 Spin coating........ 13
5.2 Self-assembly of block copolymers on hydrogel coatings........ 14
5.3 Investigation of polymer self-assembly and protein adsorption via QCM-D........ 15
5.4 Cell culture and cell sheet detachment........ 16
6. POLYMER AND SURFACE CHARACTERIZATION........ 17
6.1 Nuclear magnetic resonance (NMR) spectroscopy........ 17
6.2 Gel permeation chromatography........ 17
6.3 Spectroscopic ellipsometry (SE)........ 17
6.4 Water contact angle (CA)........ 18
6.5 Swelling of surface-bound hydrogels........ 18
6.6 Optical microscopy........ 19
7. PREPARATION AND SURFACE MODIFICATION OF PHPA-BASED BULK GELS........ 19
7.1 Synthesis of PHPA-stat-ABP-based bulk gels and surface modification with PGE-brushes........ 19
7.2 Synthesis of PHPA-MBAA bulk gels and surface modification with PGE-brushes........ 20
8. SUPPLEMENTARY IMAGES........ 21

1. MATERIALS

1.1 Materials for synthesis

Hydroxypropyl acrylate (HPA) was purchased from TCI Deutschland GmbH (Eschborn, Germany) as a mixture of 2-hydroxypropyl acrylate (2-HPA) and 1-methyl-2-hydroxyethyl acrylate (1-MeHEA) and used after filtration through Al_2O_3 to remove hydroquinone monomethyl ether (MEHQ) as the inhibitor. *N*-isopropylacrylamide (NIPAm) and 2,2'-azobis(2-methylpropionitrile) (AIBN, 98%) were supplied by Sigma Aldrich (Steinheim, Germany) and used after recrystallization in *n*-pentane:*n*-hexane (6:4) for NIPAm and methanol (MeOH) for AIBN. 4-Hydroxybenzophenone (4-HBP, 98%), triethylamine (TEA), acryloyl chloride (AAC, 97%), and *N*,*N*'-methylene bisacrylamide (MBAA), and methanol (MeOH, ≥ 99.9 %) were purchased from Sigma Aldrich and used without further purification. Dichloromethane (DCM) was supplied by Fisher Scientific (Schwerte, Germany). Sodium sulfate (Na_2SO_4, 99%) and pre-wetted regenerated cellulose dialysis tubes (molecular weight cut-off (MWCO): 3.5 kDa, Spectra/Por® 6) from SpectrumLabs were supplied by Carl Roth GmbH + Co. KG (Karlsruhe, Germany). Ethyl acetate (EtOAc) was supplied by Merck (Darmstadt, Germany). Ethanol (EtOH, technical grade) used for the synthesis as well as for surface preparation was supplied by Sigma Aldrich (Steinheim, Germany) and distilled under reduced pressure prior to use to remove impurities, while polymer stock solutions for the self-assembly process were prepared from absolute ethanol ($EtOH_{abs}$, 99%, (Fisher Scientific, Schwerte, Germany). Sodium bicarbonate ($NaHCO_3$) was supplied by Grüssing GmbH (Filsum, Germany).

1.2 Materials for surface modification

Ultrapure water for surface modification and washing was prepared via a Merck Millipore™ water treatment system Milli-Q with a minimum resistivity of 18.2 MΩ cm (25 °C). For surface characterization, silicon wafers with a 2 nm SiO_2 layer supplied by Silchem GmbH (Freiberg,

Germany) were cut into quadratic pieces (11 x 11 mm), washed with EtOH, and dried under a stream of N_2. Gold-coated QCM-D sensor chips (11 mm diameter) were supplied by Q-Sense LOT-Quantum Design GmbH (Darmstadt, Germany). The polystyrene (PS) solution (1 wt-% in toluene) used for spin-coating of the silicon wafers/gold sensors was prepared using commercial PS (M_n= 132 g/mol, $Đ$ = 1.9) from Falcon® culture dishes supplied by Th. Geyer GmbH + Co. KG (Berlin, Germany). PHPA-*stat*-ABP solutions (0.5 wt-% for 15 nm coatings, 1.5 wt-% for 50 nm coatings) in EtOH were prepared by dilution of a 2 wt.-% stock solution in EtOH. Similarly, PNIPAm-*stat*-ABP solutions (0.5 wt-% for 15 nm coatings, 2 wt-% for 100 nm coatings) in EtOH were prepared by dilution of a 2 wt-% stock solution in EtOH. Poly(glycidyl ether) (PGE) block copolymers comprising an anchor block with amide-linked, photoreactive benzophenone (BP) units were synthesized as described previously.[1] In brief, PGE-*block*-BP block copolymers were synthesised through the monomer-activated ring opening polymerization of statistically incorporated glycidyl methyl ether and ethyl glycidyl ether (1:3) monomers in the thermoresponsive block followed by allyl glycidyl ether to produce a short anchor block. Triisobutylaluminium (Al(*i*-Bu_3) served as an activator and tetraoctylammonium bromide (N(Oct_4)Br) as the initiator of the polymerization, resulting in block copolymer chains with a terminal bromine (Br) group. These Br-end groups on the polymer chains are not crucial or involved in any of the surface-directed self-assembly or reactive surface-immobilization strategies presented in this work. PGE-*block*-BP solutions were prepared by dissolving the polymer in aq. EtOH (H_2O:EtOH 48:52 v/v-%). Notably, preparing the PGE-*block*-BP solutions with freshly distilled but technical-grade EtOH (Sigma Aldrich) led to cloudiness of the solution due to residual water in the EtOH. By employing $EtOH_{abs}$, the solution appeared transparent. Therefore, all PGE-*block*-BP solutions used in this study were prepared with $EtOH_{abs}$. Phosphate buffered saline (PBS) solutions used for QCM-D analysis were prepared by dissolving PBS pellets (Sigma Aldrich, Steinheim, Germany) in

Milli-Q water. Before use, PBS solutions were sterile filtered (0.22 µm) and degassed in an ultrasonic bath for 30 min. Sodium alginate was purchased from Sigma Aldrich (#71238). Calcium chloride (97%) was purchased from Thermo Fisher Scientific (Darmstadt, Germany).

1.3 Materials for cell culture and protein adsorption studies

Falcon® PS culture dishes (Ø 35 mm) were purchased from Th. Geyer GmbH + C. KG (Berlin, Germany). Dulbecco's modified Eagle medium (DMEM) with 4.5 g L^{-1} glucose, 1% penicillin-streptomycin, trypsin/EDTA solution (0.05%), and Dulbecco's phosphate-buffered saline solution containing $CaCl_2$ and $MgCl_2$ (DPBS) were purchased from Thermo Fisher Scientific (Darmstadt, Germany). Fetal bovine serum (FBS) was purchased from PAN-Biotech GmbH (Aidenbach, Germany). Propidium iodide (PI) and fluorescein diacetate (FDA) were supplied by Sigma Aldrich (Steinheim, Germany).

2. SYNTHESIS AND CHARACTERIZATION OF 4-ACRYLOYLOXYBENZO-PHENONE (4-ABP).

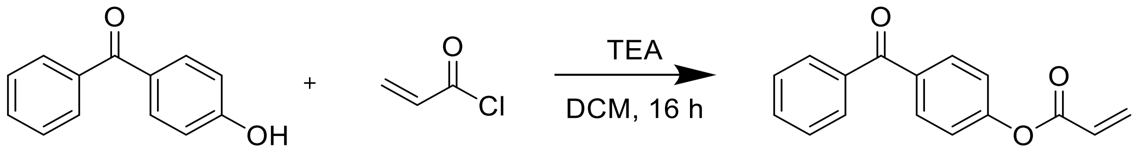

Scheme S1. Synthesis of the photo-reactive comonomer 4-ABP.

In brief, 4-HBP (5 g, 25.2 mmol) is dried in high vacuum. After dissolving 4-HBP in anhydrous DCM (20 mL) in an Ar atmosphere, TEA (4.19 mL, 30.2 mmol, 1.2 eq) is added, and the reaction mixture is cooled in an ice bath. A solution of AAC (2.44 mL, 30.2 mmol, 1.2 eq) in anhydrous DCM (10 mL) is added dropwise via a syringe over the course of 30 min at 0 °C. Afterward, the reaction is allowed to warm up to room temperature and stirred overnight without light exposure. The formed precipitate is filtered off and washed with DCM (20 mL). The organic solution is then successively washed with 0.1 M HCl (50 mL), saturated $NaHCO_3$ (50 mL), and saturated brine (50 mL). The separated organic phase is dried over Na_2SO_4,

filtered, and the filtered residue is washed with DCM. The combined organic phases are concentrated by evaporation under reduced pressure. The crude product was then purified by silica column chromatography using DCM/EtOAc (4:1) as eluent. The pure product was concentrated and obtained as a white crystalline solid in 79% yield after drying in high vacuum.

^{1}H NMR (400 MHz; $CDCl_3$): δ (ppm) = 7.81-7.72 (m, 4H, Ar-H); 7.54 - 7.39 (m, 3H, Ar-H); 7.21 - 7.18 (m, 2H, Ar-H); 6.57 (d, J= 17.3 Hz, 1H, -CH=C$\underline{H}_2$); 6.27 (dd, J= 17.3, 10.5 Hz, 1H, -C$\underline{H}$=CH_2); 5.99 (d, J=10.4 Hz, 1H, -CH=C$\underline{H}_2$); 1.58 (residual H_2O).

^{13}C NMR (101 MHz; $CDCl_3$): δ (ppm) = 195.6 (Ar-$\underline{C}$=O); 164.0 (-O$\underline{C}$=OCHCH_2); 153.9 (Ar-$\underline{C}$O); 137.5, 135.2, 132.5, 131.7, 130.0, 127.6, 121.6 (Ar-C); 133.4 (-OC=OCH$\underline{C}H_2$); 128.4 (-OC=O$\underline{C}$HCH_2).

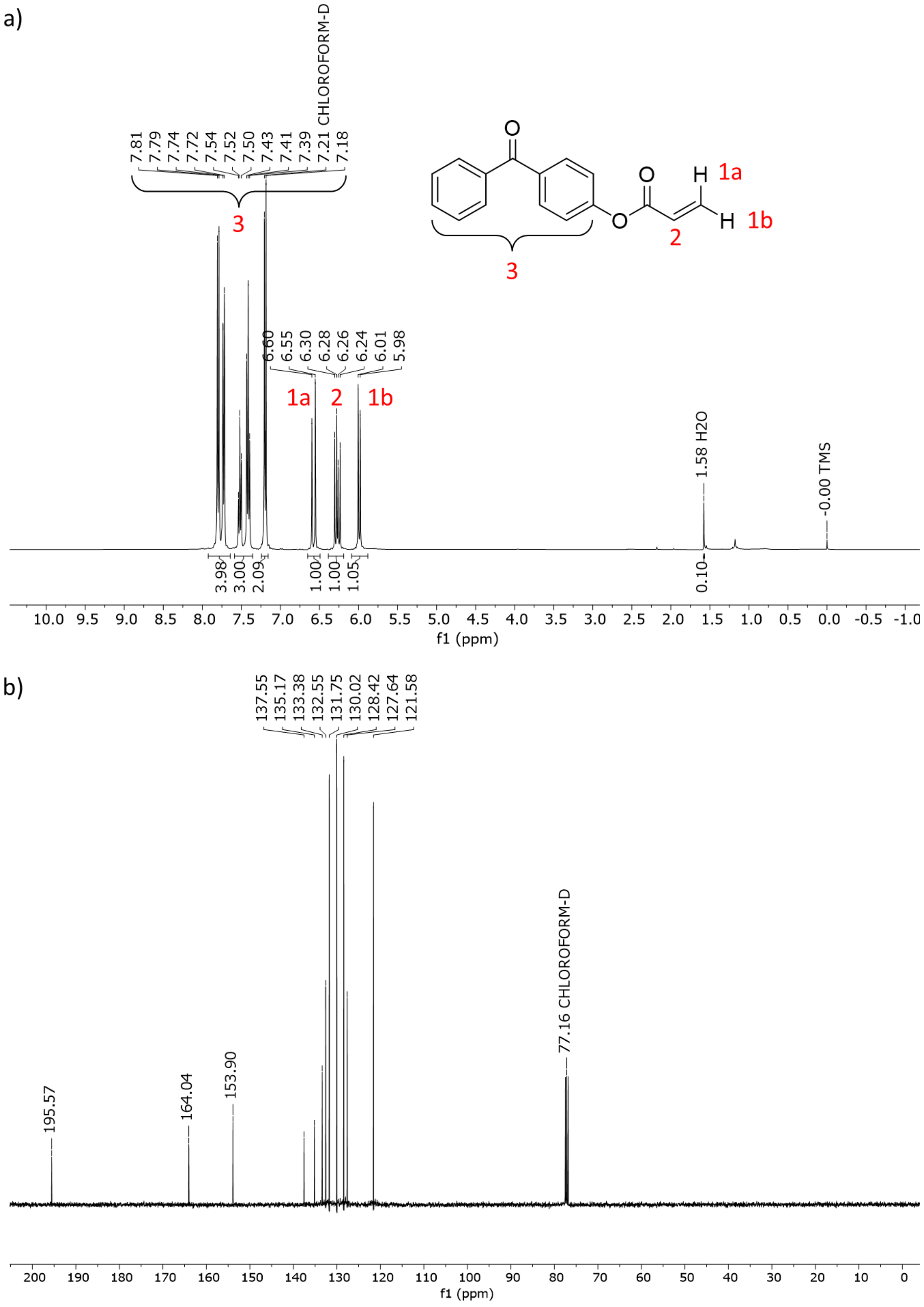

Figure S1. a) ^{1}H- and **b)** ^{13}C-NMR spectra of the photo-reactive comonomer 4-ABP recorded in $CDCl_3$ at 400 and 101 MHz, respectively.

3. SYNTHESIS AND CHARACTERIZATION OF PHPA-*stat*-ABP.

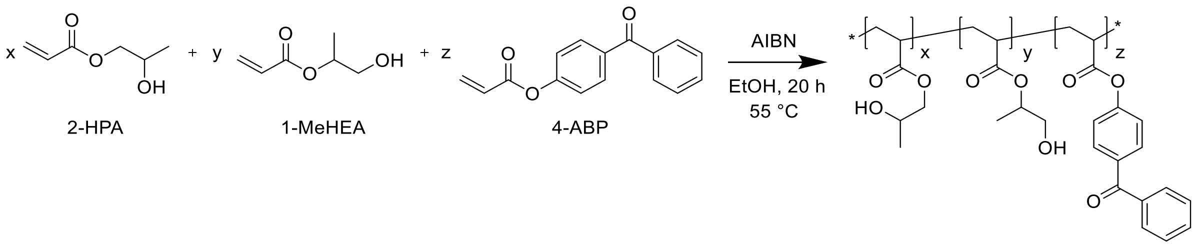

Scheme S2. Reaction scheme for the synthesis of the statistical copolymer PHPA-*stat*-ABP as a precursor for UV-triggered crosslinking and surface immobilization of PHPA-based hydrogel coatings on PS substrates. The amount of statistically incorporated BP groups in the final copolymer (51 kDa) was 2.0 mol-% as determined by ^{1}H NMR spectroscopy.

^{1}H NMR (400 MHz; $CDCl_3$): δ (ppm) = 7.80 - 7.18 (m, 9H, Ar-H); 4.92 (m, 11H, -OCO-C$\underline{H}$(-CH_3)-CH_2-OH from 1-MeHEA); 4.12 - 3.48 (m, 188 H, -OCO-C$\underline{H}_2$-C$\underline{H}$(-O$\underline{H}$)-CH_3 from 2-HPA); 3.39 (residual MeOH); 2.33 - 1.48 (m, 150 H, polymer backbone); 1.12 (s, 145 H, -OCO-CH_2-CH(-OH)-C$\underline{H}_3$ from 2-HPA).

^{13}C NMR (176 MHz, $CDCl_3$): δ (ppm) = 194.6 (Ar-$\underline{C}$O-Ar-R); 174.0 (-O$\underline{C}$O-CH_2-CH(-OH)-CH_3); 160.0 (Ar-O$\underline{C}$=OCHCH-R); 154.8 (Ar-$\underline{C}$-CO-C-Ar-R); 152.6 (Ar-C-CO-$\underline{C}$-Ar-R); 136.4 ($C_5H_5\underline{C}(CO)C_6H_4$-R); 134.4 ($C_6H_5(CO)\underline{C}C_5H_4$-R); 131.9, 130.7, 129.0, 127.4, 120.6 (Ar-C); 71.7 (-OCO-$\underline{C}$H(-CH_3)-CH_2-OH from 1-MeHEA); 69.0 (-OCO-$\underline{C}H_2$-CH(-OH)-CH_3); 64.4 (-OCO-CH_2-$\underline{C}$H(-OH)-CH_3); 57.3 (residual EtOH); 40.7 (-CH_2-$\underline{C}$H(-COO-R)-CH_2-); 34.0 (-$\underline{C}H_2$-CH(-COO-R)-CH_2); 18.0 (-OCO-CH_2-CH(-OH)-$\underline{C}H_3$ from 2-HPA); 17.4 (residual EtOH); 15.0 (-OCO-CH(-$\underline{C}H_3$)-CH_2-OH from 1-MeHEA).

GPC (THF, PS standard): M_n = 51400 Da; $Đ$ = 3.04

Table S1. Optimized conditions for synthesizing high molecular weight PHPA-*stat*-ABP via the free-radical polymerization in ethanol initiated by 0.5 mol-% azobis(iso butyronitrile).

**Polymer**	**HPA [wt-%]**	**ABP[a] [mol-%]**	***T* [°C]**	**Yield[b] [%]**	**M_n[c] [kDa]**	**Đ[c]**
PHPA-*stat*-ABP	40	2.0	55	86	51.4	3.04

[a] Calculated from ^{1}H-NMR spectrum. [b] Calculated after dialysis of the crude polymer against MeOH for 3 days, isolation, and drying. [c] Determined from GPC measurements in THF applying PS standards.

The content of incorporated ABP (mol-%) in the PHPA-*stat*-ABP copolymer was calculated from the ^{1}H-NMR spectrum by first referencing the peak integral of the aromatic protons between 7.80 – 7.70 ppm to 4 protons (corresponding to a fraction of the aromatic BP protons). The ratio of the integral value $I_{7.80}$ of the aromatic signals and the integral $I_{2.33}$ corresponding to the 3 protons per monomer unit of the polymer backbone between 2.33 – 1.48 ppm yields the mol-% of incorporated ABP, according to the following equation.

$$\text{ABP mol-\%} = [(I_{7.80}/4) \times (3 / I_{2.33})] \times 100$$

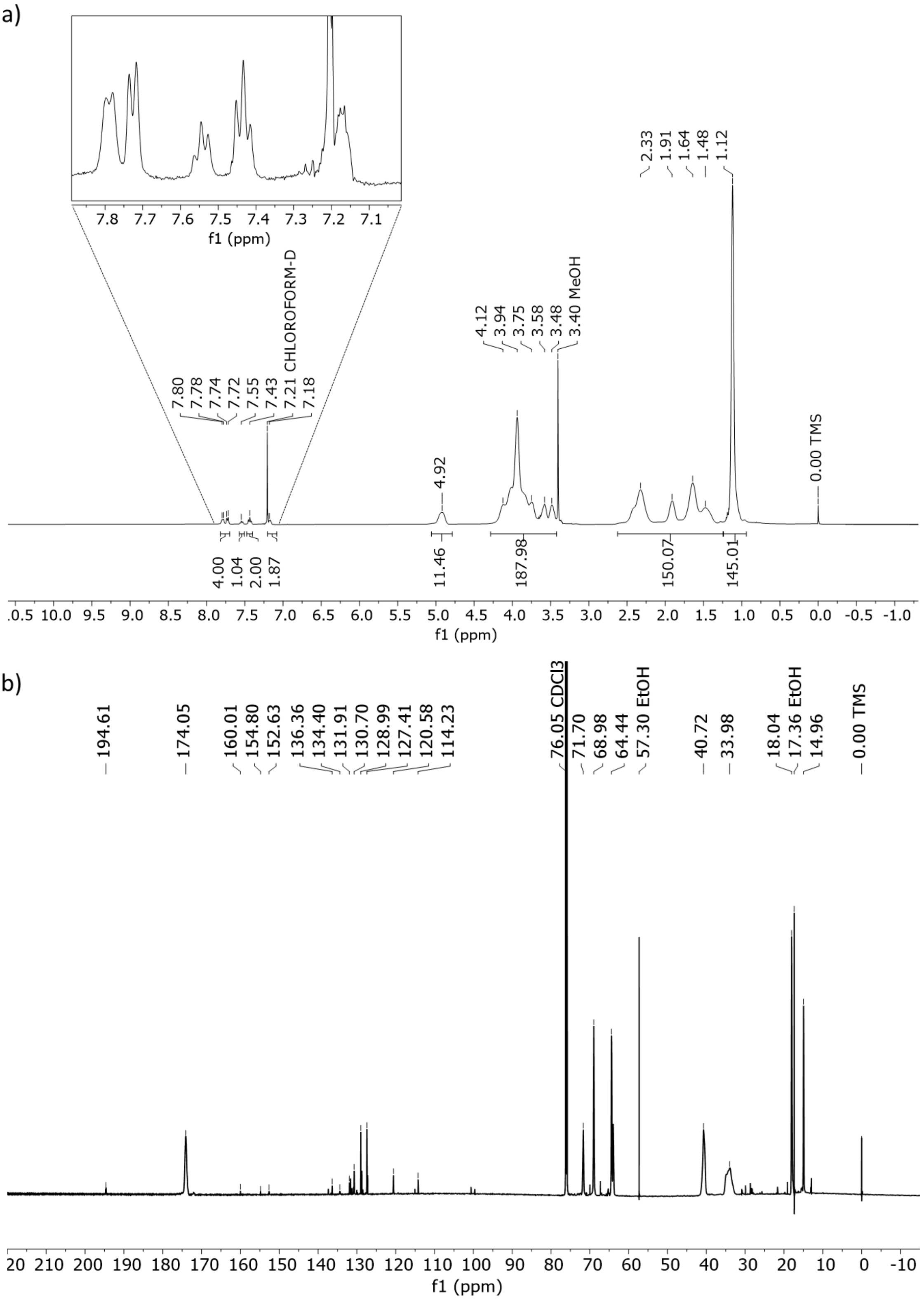

Figure S2. a) ^{1}H- and **b)** ^{13}C-NMR spectra of the statistical copolymer PHPA-*stat*-ABP with 2 mol-% ABP recorded in $CDCl_3$ at 400 and 176 MHz, respectively.

4. SYNTHESIS AND CHARACTERIZATION OF PNIPAm-*stat*-ABP.

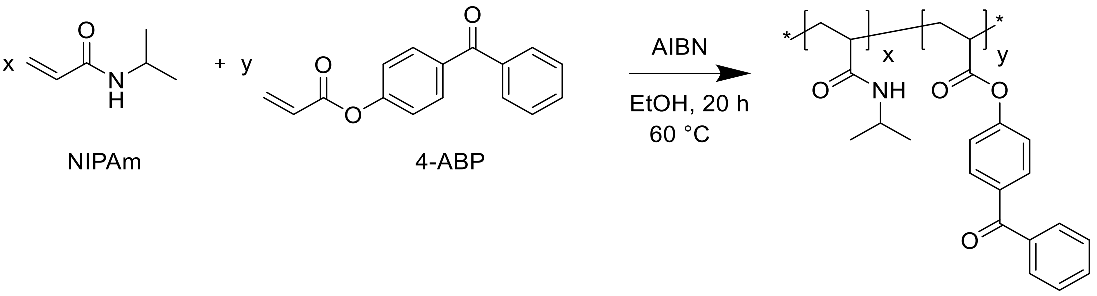

Scheme S3. Reaction scheme for the synthesis of the statistical copolymer PNIPAm-*stat*-ABP as a precursor for UV-triggered crosslinking and surface immobilization of PNIPAm-based hydrogel coatings on PS substrates. The amount of statistically incorporated BP groups in the final copolymer (62 kDa) was 2.4 mol-% as determined by ^{1}H NMR spectroscopy.

^{1}H NMR (400 MHz; $CDCl_3$): δ (ppm) = 7.75 - 7.42 (m, 9H, Ar-H); 6.72 (m, 30H, -CO-N$\underline{H}$-CH(-CH_3)$_2$); 3.93 - 3.83 (m, 88 H, -CO-NH-C$\underline{H}$(-CH_3)$_2$); 3.65 (residual EtOH); 2.11 - 1.61 (m, 125 H, polymer backbone); 1.08 (s, 236 H, -CO-NH-CH(-C$\underline{H}_3$)$_2$).

^{13}C NMR (151 MHz, $CDCl_3$): δ (ppm) = 194.6 (Ph-$\underline{C}$O-Ph); 173.0 (-$\underline{C}$O-NH-CH(CH_3)$_2$); 152.9 (Ar-$\underline{C}$-CO-Ar); 136.4 ($C_5H_5\underline{C}$(CO)C_6H_4-R); 134.1 (C_6H_5(CO)$\underline{C}C_5H_4$-R); 131.5, 130.6, 128.9, 127.3, 120.6 (Ar-C); 57.0 (residual EtOH); 49.1 (residual MeOH); 41.5 (-CO-NH-$\underline{C}$H-(CH_3)$_2$); 40.3 (-CH_2-$\underline{C}$H(-CO-NH-CH-(CH_3)$_2$); 35.5 (-$\underline{C}H_2$-CH(-CO-NH-CH-(CH_3)$_2$); 21.6 (-CO-NH-CH($\underline{C}H_3$)$_2$); 17.5 (residual EtOH).

GPC (DMF, PS standard) = 62270 Da; $Đ$ = 3.63

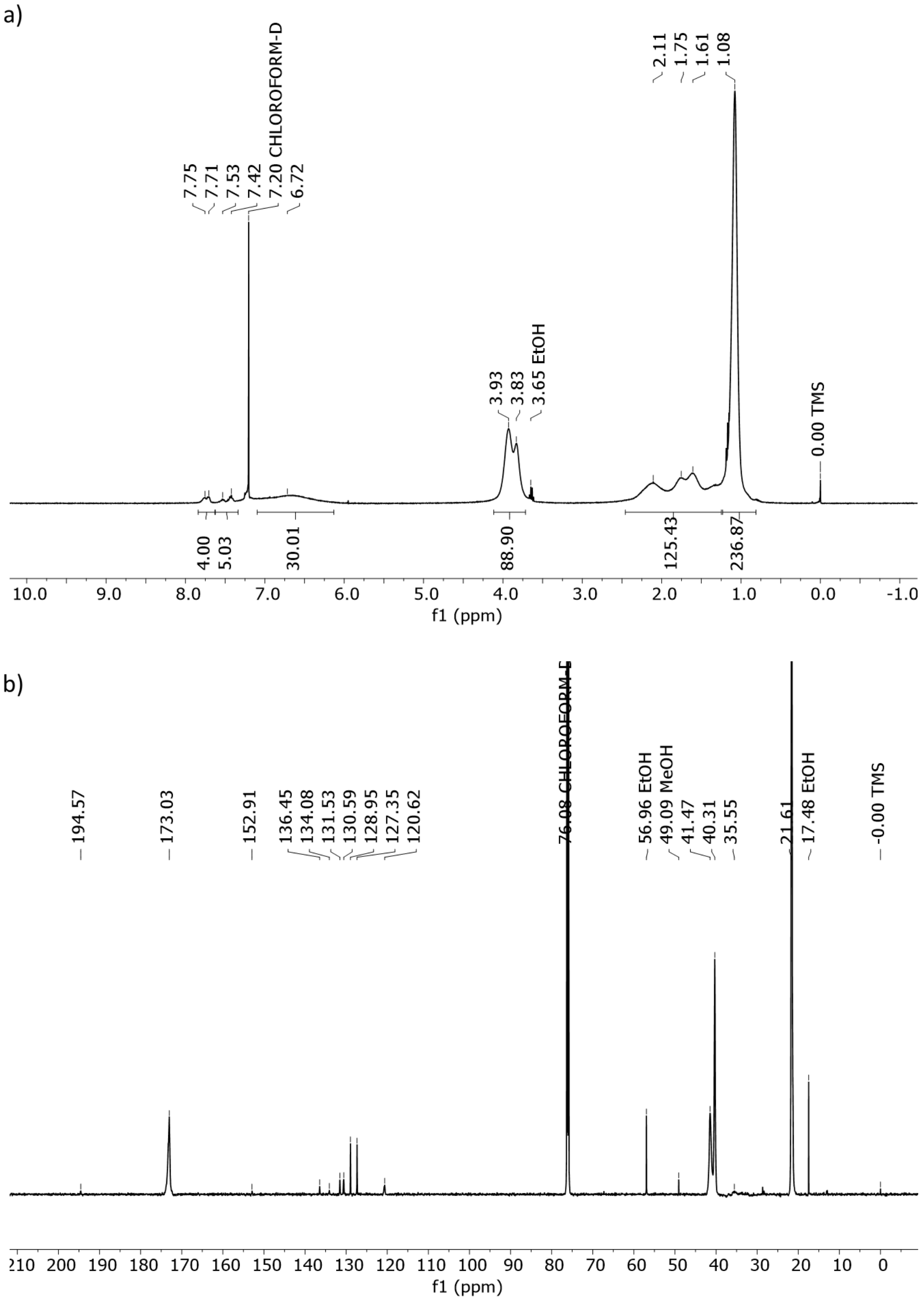

Figure S3. a) ^{1}H and **b)** ^{13}C-NMR spectra of the statistical copolymer PNIPAm-*stat*-ABP with 2 mol-% ABP recorded in $CDCl_3$ at 400 and 151 MHz, respectively.

The content of incorporated ABP (mol-%) in the PNIPAm-*stat*-ABP copolymer was calculated from the ^{1}H-NMR spectrum by first referencing the peak integral of the aromatic protons between 7.75 – 7.71 ppm to 4 protons (corresponding to a fraction of the aromatic BP protons). The ratio of the integral value $I_{7.75}$ of the aromatic signals and the integral $I_{2.11}$ corresponding to the 3 protons per monomer unit of the polymer backbone between 2.11 – 1.61 ppm yields the mol-% of incorporated ABP, according to the following equation.

$$\text{ABP mol-\%} = [(I_{7.75}/4) \times (3 / I_{2.11})] \times 100$$

5. METHODS FOR SURFACE MODIFICATION AND CHARACTERIZATION

5.1 Spin coating.

Spin-coating of thin PS films on silicon substrates was performed using a spin coater (WS-650-23) from Laurell Technologies Corporation (North Wales, PA, USA), applying a drop (50 µL) of a PS solution (1 wt-%) in toluene at 3000 rpm for 60 s. Gold sensors for quartz crystal microbalance measurements were coated similarly at 3000 rpm for 60 s using 30 µL of a PS solution (1 wt-%) in toluene. PS-coated silicon, as well as gold substrates, were subsequently dried in vacuum at 400 mbar and 60 °C for 2 h. PHPA-*stat*-ABP-based hydrogels on PS-coated silicon and gold substrates were prepared by spin-coating 50 µL of the copolymer solution (0.5 wt-% for ~15 nm coatings, 1.5 wt-% for ~50 nm coatings) in EtOH. PNIPAm-*stat*-ABP-based hydrogels on PS-coated silicon substrates were prepared by spin-coating 50 µL of the copolymer solution (0.5 wt-% for ~15 nm coatings, 2 wt-% for ~100 nm coatings) in EtOH. Alginate coatings on silicon wafers were prepared by spin coating 50 µL of a 1 wt-% sodium alginate solution at 3000 rpm for 120 s and crosslinking *in situ* by dropping a 3 wt-% $CaCl_2$ solution after 15 s of spinning. The alginate-coated silicon wafers were then placed in Milli-Q water on a shaking plate for 3 h, rinsed with Milli-Q water, and dried at 80 °C. For the preparation of PHPA-*stat*-ABP as well as PNIPAm-*stat*-ABP-based hydrogels on suspension

culture dishes (Falcon® PS Petri dishes; Ø 3.5 cm), the dishes were similarly spin-coated at 3000 rpm for 60 s by applying 100 µL of the 0.5 wt-% polymer solution in EtOH. Covalent immobilization, as well as cross-linking of the spin-coated BP-containing polymers on silicon, gold as well as cell culture substrates, was achieved through UV-light irradiation using a UV-KUB 2 from Kloè (Montpellier, France) with a wavelength of 365 nm and an intensity of 25 mW cm^{-2} (100%) for 320 s. The hydrogel-coated substrates were extracted in EtOH for 18 h, subsequently rinsed with Milli-Q water, and used for further experiments after gentle drying under a stream of N_2.

5.2 Self-assembly of block copolymers on hydrogel coatings.

PGE-*block*-BP brush coatings on PHPA-*stat*-ABP hydrogels were prepared statically by incubating the hydrogel-coated substrates in 2 mL of the PGE-*block*-BP copolymer solution (250, 125, 62.5, 31.25 µg mL^{-1} for silicon wafers, 250 µg mL^{-1} for Petri dishes, 300 µg mL^{-1} for gold substrates) in aq. EtOH for 1 h in the absence of light at room temperature (RT) (20 °C). Similarly, PGE-*block*-BP brush coatings on PNIPAm-*stat*-ABP hydrogels were prepared statically by incubating the hydrogel-coated substrates in 2 mL of the PGE-*block*-BP copolymer solution (250 µg mL^{-1} for silicon wafers and Petri dishes) in aq. EtOH for 1 h in the absence of light. After gently discarding the supernatant solution from the hydrogel samples, the surfaces were carefully dried under a stream of N_2 and subsequently irradiated with UV light (UV-KUB 2; 365 nm; 25 mW cm^{-2}) for 320 s. The so-prepared bilayer structures were extracted in EtOH until the dry layer thickness measured by SE remained constant (~ 5-10 h). For subsequent CA, SE, quartz crystal microbalance with dissipation monitoring (QCM-D), and cell culture experiments, the bilayer coatings were washed with Milli-Q water and dried under a stream of N_2. The dry thickness of PGE-*block*-BP brush coatings on PHPA-*stat*-ABP and PNIPAm-*stat*-ABP hydrogels was determined similarly to the dry thickness of bare hydrogel coatings by adding a Cauchy layer with a fixed refractive index $n = 1.45$ on top of the

described model above (see section *Spectroscopic Ellipsometry*) and air as the surrounding medium. The PGE-*block*-BP concentration in the self-assembly solution was slightly increased from 250 to 300 µg mL^{-1} to obtain comparable brush layer thicknesses of the PHPA-based bilayers **B4** on QCM-D gold sensors and silicon substrates.

5.3 Investigation of polymer self-assembly and protein adsorption via QCM-D.

For dynamic online (real-time) adsorption experiments, QCM-D measurements were performed with a one-channel Q-Sense E1 device from LOT-Quantum Design GmbH (Darmstadt, Germany) equipped with a Reglo Digital peristaltic pump from Ismatec (Wertheim, Germany). The software QSoft401 version 2.5.22 was used for data acquisition, and QTools 3 version 3.1.25 from Biolin Scientific AB (Stockholm, Sweden) was used for data analysis. AT-cut crystals with a fundamental resonance frequency of 4.95 MHz were mounted in a standard flow module (Biolin Scientific AB, Stockholm, Sweden) with the polymer-coated side exposed to the flow chamber. The temperature was controlled within ± 0.1 °C for all experiments. The mass sensitivity constant C of the sensor was 17.7 ng cm^{-2} Hz^{-1} and was used to convert detected frequency changes Δf on the sensor chip into mass changes Δm via the Sauerbrey equation. All the results in the present study are obtained from the evaluation of the frequency change in the third overtone ($f_n = 3$).

For polymer adsorption, preformed PHPA-*stat*-ABP hydrogels on PS-coated QCM-D gold sensors showing a dry layer thickness of 13.6 ± 0.2 nm via SE (n = 3) were used and equilibrated in aq. EtOH in the QCM-D at 20 °C. Then, dilute solutions (250 µg mL^{-1}) of PGE (M_n = 26 kDa, $Đ$ = 1.08) lacking the BP-based anchor block as well as PGE-*block*-BP copolymers in aq. EtOH were passed over the hydrogel-coated sensors under dynamic conditions (0.1 mL min^{-1}) for 10 min, followed by a switch to aq. EtOH for ~ 20 min to remove non-adsorbed polymers until Δf reached a plateau. For protein adsorption measurements on the hydrogel- as well as bilayer-coated sensors, DMEM cell culture medium supplemented with

10% FBS was used as protein-containing medium. The solution was injected into the chamber after establishing a stable baseline with pre-warmed PBS buffer at either 20 or 37 °C under a constant flow of 0.1 mL min^{-1}. After 20 min of protein-containing medium flow, the system was rinsed with PBS to remove loosely adsorbed proteins until the frequency shift reached a stable plateau again.

5.4 Cell culture and cell sheet detachment.

Human dermal fibroblasts (HDFs) were cultured, as reported previously, in DMEM cell culture medium supplemented with 10% FBS and 1% penicillin-streptomycin (DMEM+) in a humified atmosphere at 37 °C and 5% CO_2.[2] For passaging, cells were trypsinized (0.05% trypsin-EDTA) for 5 min at 37 °C followed by trypsin inhibition by addition of cell culture medium. Cells were centrifuged at 140*g* for 4 min, and the supernatant was aspirated. The cell pellet was resuspended in DMEM+, and cells were used in passages 3 to 7 for the experiments. Polymer-coated petri dishes for cell culture were disinfected with 70% EtOH for 10 min under the sterile bench and subsequently washed twice with cold DPBS. The sterile dishes were then either used directly after disinfection or stored in sterile conditions in the absence of light for a maximum of 7 days before use. For the polymer-coated dishes that underwent pre-incubation with cell culture medium, 2 mL of DMEM+ was applied to the dishes at 37 °C and 5% CO_2 for 30 min. Polymer-coated dishes and TCPS controls were seeded with 3.15×10^5 cells in 2 mL cell culture medium per dish (3.5×10^4 cells cm^{-2}) and cultured (37 °C, 5% CO_2) for the indicated time with frequent media changes every 2-3 days. Cells were observed via phase contrast microscopy after 4, 24, 48, and 72 h. For live/dead staining of HDFs on PHPA-based bilayers, a staining solution containing 50 µM PI and 10 µM FDA was prepared in DMEM. After aspiration of the medium, the cells were rinsed with DPBS and incubated in staining solution (2 mL) at 37 °C for 5 min. After aspiration of the staining solution, samples were imaged in DPBS in fluorescent mode with appropriate filter sets. For temperature-induced detachment on

PHPA-based bilayers, HDFs (1.35 x 10^5 cells cm^{-2}) were seeded onto non-pre-treated dishes. After 24 h culture at 37 °C and 5% CO_2, confluent cell dishes were taken out of the incubator and placed on the bench at RT until complete detachment of intact sheets.

6. POLYMER AND SURFACE CHARACTERIZATION

6.1 Nuclear magnetic resonance (NMR) spectroscopy.

^{1}H and ^{13}C NMR spectra were recorded either on a Jeol ECX at 400 and 100 MHz, respectively, a Bruker AVANCE at 700 and 176 MHz, respectively, or a Jeol ECZ at 600 and 151 MHz, respectively. All spectra were processed with the software MestReNova (version 14.2.2). Chemical shifts (δ) were reported in ppm after referencing the singlet of the internal standard tetramethyl silane at $\delta = 0$ ppm.

6.2 Gel permeation chromatography.

GPC measurements of PHPA-*stat*-ABP were carried out in THF as eluent at a concentration of 6 mg mL^{-1} and a flow rate of 1 mL min^{-1} at 25 °C. Calibration was established with PS standards from PSS (Mainz, Germany) and the PSS Win-GPC software. Three PLgel mixed-C columns (Agilent, Waldbronn, Germany) with dimensions of 7.5 x 300 mm and a particle size of 5 µm were used in line with a refractive index detector. GPC measurements on PNIPAm-*stat*-ABP were performed using dimethylformamide (DMF) containing 3 mg mL^{-1} lithium bromide and 6 mg mL^{-1} acetic acid as eluent at a concentration of 10 mg mL^{-1} and a flow rate of 1 mL min^{-1} at 40 °C. Calibration was established with PS standards from PSS (Mainz, Germany) and the LabSolutions software. Three PSS PolarSil analytical columns (1000, 300, and 100 Å) with dimensions 8 x 300 mm and a particle size of 5 µm were used in line with a refractive index detector.

6.3 Spectroscopic ellipsometry (SE).

For the characterization of PS films, the dry layer thickness, as well as the refractive index, were measured by SE at an incident angle of 70° and wavelengths from 370 to 1070 nm with a SENpro spectroscopic ellipsometer from SENTECH Instruments GmbH (Berlin, Germany) and calculated as an average value of five different spots on the surface and further used as fixed values for the subsequent modelling of the hydrogel layer. The dry thickness of PHPA-*stat*-ABP, as well as PNIPAm-*stat*-ABP hydrogel layers, was determined similarly as average of five different spots on the surface by fitting a model consisting of a silicon dioxide layer, a PS layer with fixed parameters, and a Cauchy layer – the layer to be determined – with a fixed refractive index $n = 1.45$ (PHPA-*stat*-ABP) and $n = 1.48$ (PNIPAm-*stat*-ABP) and air as the surrounding medium.

6.4 Water contact angle (CA).

The wettability of the coatings was determined by static CA measurements with an OCA contact angle system from DataPhysics Instruments GmbH (Filderstadt, Germany) and fitted with the software package SCA202 (version 3.12.11) using the sessile drop configuration. CAs of the immobilized hydrogels were determined after extraction of non-crosslinked chains at 20 °C (PHPA-*stat*-ABP and PNIPAm-*stat*-ABP) and 37 °C (PNIPAm-*stat*-ABP). Therefore, a drop of Milli-Q water (2 µL) was placed onto the surface, and CAs were determined right after deposition with the Young-Laplace model or, if indicated, 5s after equilibration. For each substrate, CAs were measured on five different spots to test for sample homogeneity and at least four independent substrates ($n \geq 4$) to test for reproducibility.

6.5 Swelling of surface-bound hydrogels.

The swelling behavior of surface-bound hydrogels was assessed in water and aqueous ethanol (48 v/v-% H_2O) as the selective solvent used in subsequent brushing-up experiments. Therefore, PHPA-*stat*-ABP and PNIPAm-*stat*-ABP gel coatings with a dry thickness of 44.3 ± 0.5 nm and 105.7 ± 4.6 nm ($n = 3$), respectively, were prepared on PS-functionalized silicon

wafers. The swelling ratio of PHPA-*stat*-ABP and PNIPAm-*stat*-ABP was evaluated in Milli-Q water and in the H_2O:EtOH mixture (48:52 v/v-%) by SE. The swelling ratio was calculated according to Equation S1 (eq S1),

$$SR = (d_{wet} - d_{dry})/d_{dry} \quad \text{(eq. S1)}$$

where d_{wet} and d_{dry} correspond to the thickness of the wet and dry gel coatings. The wet thickness of gel coatings was measured by SE in the fluid chamber at RT after equilibration in both solvents and normalized to their dry thickness. For the liquid measurements, n = 1.333 and n = 1.356 were used as refractive indices of the medium for fitting the SE model in water and aqueous ethanol, respectively. Fitting of such model yielded n = 1.42 ± 0.01 and n = 1.40 for respectively PHPA-*stat*-ABP and PNIPAm-*stat*-ABP coatings in water, and n = 1.40 and n = 1.43 in aqueous ethanol.

6.6 Optical microscopy.

Microscopic images of human dermal fibroblasts (HDFs) were taken on a Zeiss Observer Z1 from Carl Zeiss Microscopy GmbH (Jena, Germany) and processed with the software Zen 3.5 version 3.5.093.00001.

7. PREPARATION AND SURFACE MODIFICATION OF PHPA-BASED BULK GELS

7.1 Synthesis of PHPA-*stat*-ABP-based bulk gels and surface modification with PGE-brushes.

PHPA-*stat*-ABP-based bulk hydrogels were prepared from a solution of 500 mg mL^{-1} PHPA-*stat*-ABP in EtOH. The solution was placed in a self-casted mask (46 mm x 14 mm x 4 mm (L x W x H)) made from medical grade silicone consisting of four independent compartments (7 mm x 7 mm x 4 mm). The silicone mask was placed on a microscope glass coverslip, and each compartment was filled with 250 µL PHPA-*stat*-ABP solution. Subsequently, the samples were irradiated with UV light (UV-KUB 2; 365 nm; 25 mW cm^{-2}) for 13 min from the upper side.

Then, the silicone mask containing the gelled samples was flipped upside down and irradiated for an additional 13 min to avoid a crosslinking gradient throughout the samples. The so-prepared PHPA-*stat*-ABP bulk gels were carefully removed from the silicone mask and placed in a 1:1 EtOH:H_2O mixture overnight to extract uncrosslinked chains. After that, the samples were kept overnight in Milli-Q water and used for further analysis/functionalization.

After gently drying the outer surface of PHPA-based gel samples with a stream of N_2, PGE-*block*-BP brush coatings on PHPA-*stat*-ABP bulk gels were prepared by incubating the gel substrates in 3 mL of the PGE-*block*-BP copolymer solution (250 μg mL^{-1}) for 1 h in the absence of light. After incubation, the samples were gently dried under a stream of N_2 and directly irradiated with UV light (UV-KUB 2; 365 nm; 25 mW cm^{-2}) for 320 s. As a control, pristine, non-functionalized PHPA-*stat*-ABP gel samples were parallelly irradiated for 320 s to exclude a change in gel surface properties due to irradiation. Samples were then extracted in a 1:1 EtOH:H_2O mixture overnight, rinsed with Milli-Q water, and stored in Milli-Q water before further analysis.

7.2 Synthesis of PHPA-MBAA bulk gels and surface modification with PGE-brushes.

For the free radical crosslinking polymerization of PHPA-MBAA bulk gels, the crosslinker MBAA (0.133 g, 8.66 x 10^{-4} mol, 3 mol-% with respect to the monomer) was placed in a glass vial together with 1 mol-% AIBN (0.0463 g, 2.82 x 10^{-4} mol) and dissolved in 10.75 mL MeOH (70 wt-%). Then, HPA (3.65 g, 2.8 x 10^{-2} mol) was added to the reaction mixture. Subsequently, the pre-polymer solution was sealed with a septum and degassed via N_2 flushing through the solution for 30 min. The reaction was initiated by placing the vial in a pre-heated oil bath at 50 °C for 24 h. After gelation, the samples were washed iteratively with acetone and water (24 h) to remove unreacted starting material. After swelling in Milli-Q water overnight, the gel samples were cut into four pieces and used for further functionalization and analysis.

After drying the outer surface of PHPA-MBAA gels with a stream of N_2, PGE-*block*-BP brush coatings on PHPA-MBAA gels were prepared by incubating the bulk gel substrates in 3 mL of the block copolymer solution (300 µg mL^{-1}) in aq. EtOH (48 v/v-% H_2O) for 1.5 h in the absence of light. A higher PGE-*block*-BP concentration and a longer incubation time were chosen to account for the larger surface area of the PHPA-MBAA gels with respect to the bulk PHPA-*stat*-ABP-based gels (see section *Self-assembly of block copolymers on hydrogel coatings*). After incubation, the samples were gently dried under a stream of N_2 and directly irradiated with UV light (UV-KUB 2; 365 nm; 25 mW cm^{-2}) for 5 min each from the top and bottom side by flipping the samples upside down after 5 min. As a control, pristine, non-functionalized PHPA-MBAA gel samples were parallelly irradiated for 5 min each from top and bottom to exclude a change in gel surface properties due to irradiation. Samples were then extracted in EtOH overnight, rinsed with Milli-Q water, and stored in Milli-Q water before further analysis.

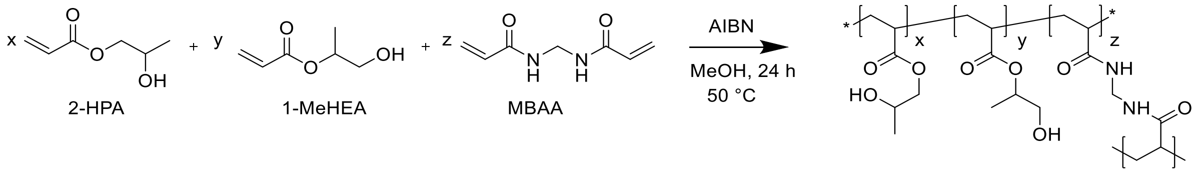

Scheme S4. Synthesis of the copolymer PHPA-MBAA with in situ crosslinking in the presence of 3 mol-% MBAA through free radical polymerization.

8. SUPPLEMENTARY IMAGES

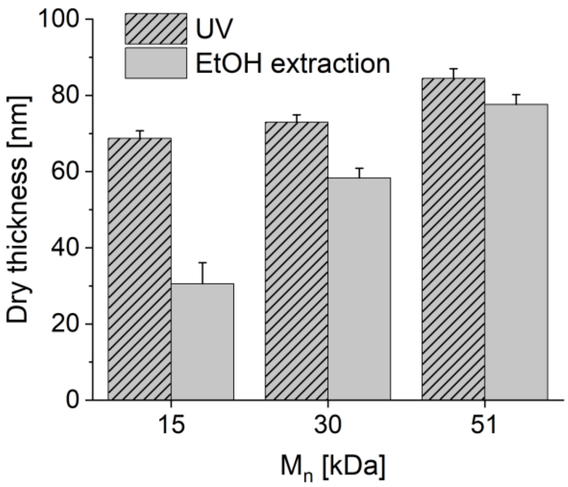

Figure S4. Dry layer thickness of PHPA-*stat*-ABP hydrogels after UV-immobilization (patterned) and extraction in ethanol (solid). The gel coatings were produced from statistical PHPA-*stat*-ABP copolymers with M_n of 15.4, 29.8, and 51.4 kDa containing 2 mol-% of the photoreactive comonomer 4-ABP on PS-coated silicon wafers substrates. Polymers with molecular weights of 15.4 and 29.8 kDa were excluded from this study due to inefficient network formation, as indicated by the relatively large loss in thickness after UV exposure and extraction. Error bars indicate the standard deviation. (n = 4)

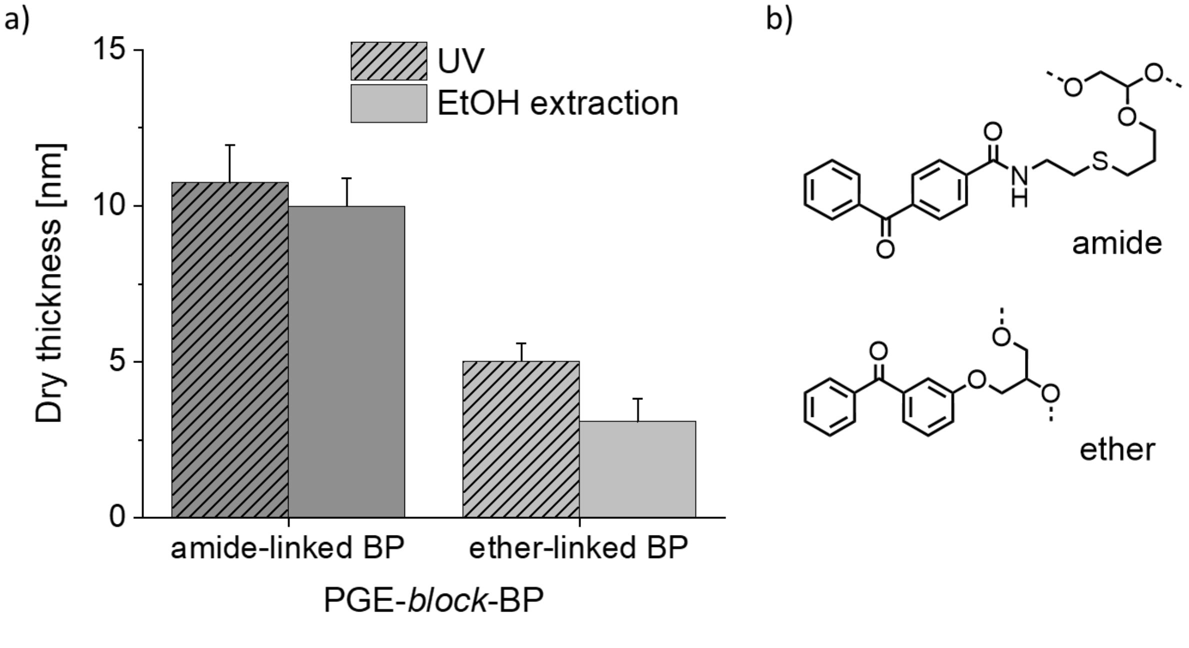

Figure S5. **a)** Dry layer thickness after UV-immobilization (patterned) and extraction in ethanol (solid) of self-assembled brushes on PHPA-*stat*-ABP hydrogel coatings with PGE-*block*-BP copolymers at a concentration of 250 µg mL^{-1} at RT (20 °C) for 1 h comprising amide and ether linkages between the BP unit and the anchor block backbone and **b)** their respective chemical structures. Block copolymers lacking the amide groups do not efficiently self-assemble from selective solvents on the hydrophilic hydrogel substrates. Layer thicknesses were measured via SE, error bars indicate the standard deviation. (n = 3)

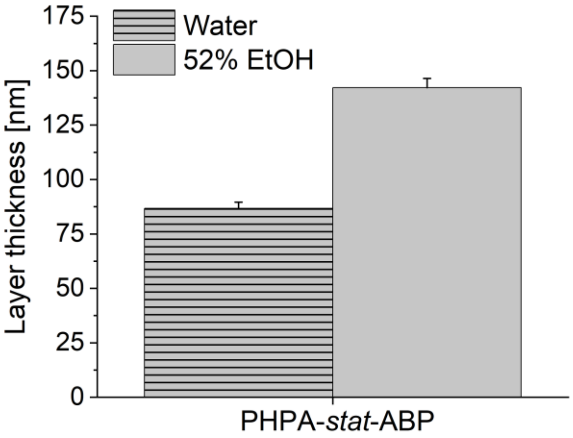

Figure S6. Solvated layer thickness of PHPA-*stat*-ABP hydrogel coatings on PS in water (patterned) or aqueous ethanolic solution (48:52 v/v-%) (solid) as the selective solvent used during brush self-assembly assessed via SE in the respective medium at 20 °C. The results clearly indicate that the hydrogel, despite its thermoresponsiveness in water, is in the swollen solvated state during the copolymer self-assembly process in the applied selective solvent. Error bars indicate the standard deviation. (n = 3)

Calculation of the brush grafting density (*GD*):

$$GD = \frac{\delta \, d_h \mathrm{N_A}}{M_n} \qquad \text{(eq. S2)}$$

where δ corresponds to the bulk density of PGE-*block*-BP (1.2 g cm^{-3}, approximated as for poly (ethylene glycol)),[3,4] d_h is the dry thickness of the immobilized brushes on PHPA-*stat*-ABP after extraction, N_A is Avogadro's number, and M_n= 28100 g mol^{-1}.

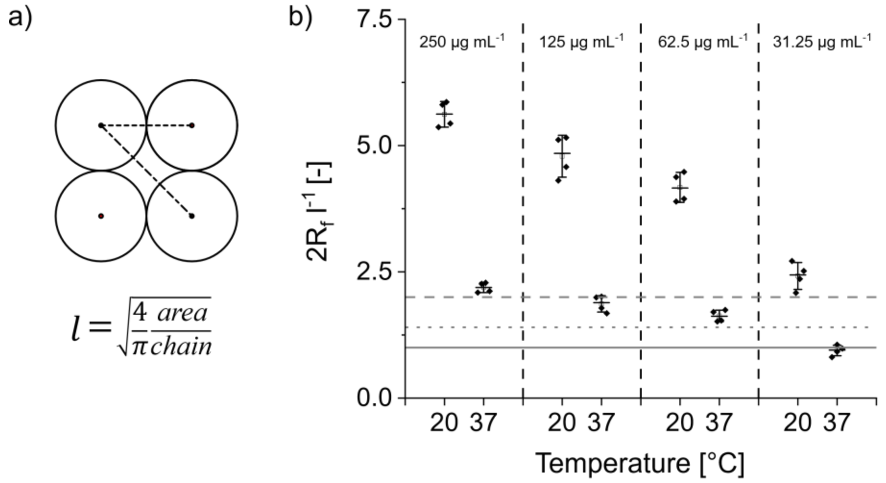

Figure S7. Estimating surface coverage of brushed-up hydrogels with PGE block copolymers exhibiting an average molecular weight M_n = 30 kDa. **a)** Schematic representation of surface area projections of surface tethered globular polymer chains on a surface with the dashed lines depicting the anchor distance *l* between neighbouring grafting sites and the equation relating the anchor distance *l* between directly neighbouring polymer chains to their grafting density.[5] **b)** Calculated degree of chain overlap $2R_f\, l^{-1}$ (R_f = Flory radius;[6,7] *l* = anchor distance[8] calculated according to the geometrical representation depicted in (**a**) at the respective GDs) of self-assembled PGE brushes on PHPA-*stat*-ABP hydrogel coatings with varying PGE-*block*-BP concentrations. The Flory radius R_f of the PGE copolymer was estimated from the number of the repeating units (286) and an estimated monomer length a = 0.37 nm. [1,2,9] Water at 20 °C

can be assumed as a theta solvent for PGE ($R_f = a\ N^{1/2}$), while at 37 °C, due to the thermoresponsiveness of PGE, water is considered a bad solvent (R_f = a $N^{1/3}$), impacting the calculation of the Flory radius. Horizontal lines represent the start of the chain overlap regime ($2R_f\ l^{-1} \geq 1$, solid line), full surface coverage ($2R_f\ l^{-1} \geq 1.4$, short dashed line), and the extended brush regime ($2R_f\ l^{-1} \geq 2$, dashed line). The calculated degree of chain overlap is plotted for each measured replicate with their mean value (cross) and SD (whiskers). Starting from a PGE-*block*-BP concentration of 62.5 µgmL^{-1}, the self-assembly process produces brushed-up gels in the brush regime, fully covering the gel surface both above and below the phase transition temperature of the grafted PGE. (n = 4)

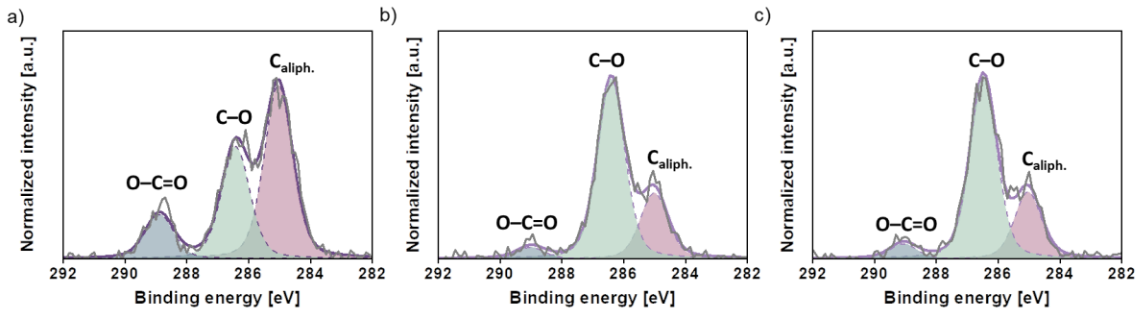

Figure S8. Highly resolved C1s XP spectra (gray line) of the **a)** PS-immobilized PHPA-*stat*-ABP gel (~50 nm dry thickness), **b)** PS-immobilized PHPA-*stat*-ABP gel (~50 nm dry thickness) with ~5 nm PGE brush coating and the **c)** PS-immobilized PHPA-*stat*-ABP gel (~15 nm dry thickness) with 5 nm PGE brush coating (**B2**). The fitted peak components and the sum curve of all fitted peak components are indicated by the purple dashed and solid lines, respectively.

Table S2. XPS fitting parameters, binding energies, and peak assignments of the bare PS substrate, PS-immobilized PHPA-*stat*-ABP gels (**H**), PS-immobilized PHPA-*stat*-ABP gel (~50 nm), PS-immobilized PHPA-*stat*-ABP gel with PGE-*block*-BP coating (**B4**), PS-immobilized PHPA-*stat*-ABP gel with thinner PGE-*block*-BP coating (**B2**), PS-immobilized PHPA (~50 nm) with PGE-*block*-BP coating (~5 nm).

Sample	Spectrum	Binding energy	L-G Mixing[a]	FWHM[b]	Assignment	Abs. Area	Relat. Area
PS substrate	C1s	284.6	0.3	0.7	C–C $_{aromatic}$	312	0.88
		285.0	0.3	0.7	C–C $_{aliphatic}$	42	0.12
PS-immobilized PHPA (**H**) (~15 nm)	C1s	284.1	0.3	1.0	charge	9	0.03
		285.0	0.3	1.0	C–C $_{aliphatic}$	142	0.52
		286.3	0.3	1.0	C–O	86	0.31
		288.8	0.3	1.0	O–C=O	38	0.14
PS-immobilized PHPA (~50 nm)	C1s	285.0	0.3	1.1	C–C	150	0.53
		286.4	0.3	1.1	C–O	97	0.34
		288.9	0.3	1.1	O–C=O	38	0.13
PS-immobilized PHPA with PGE coating (~10 nm, **B4**)	C1s	285.0	0.3	1.0	C–C	77	0.28
		286.3	0.3	1.0	C–O	192	0.69
		289.0	0.3	1.0	O–C=O	8	0.03
PS-immobilized PHPA (~50 nm) with thinner PGE coating (~5 nm)	C1s	285.0	0.3	1.0	C–C	73	0.25
		286.5	0.3	1.0	C–O	200	0.69
		289.0	0.3	1.0	O–C=O	16	0.06
PS-immobilized PHPA with thinner PGE coating (~5 nm, **B2**)	C1s	285.0	0.3	1.0	C–C	72	0.26
		286.4	0.3	1.0	C–O	198	0.70
		289.0	0.3	1.0	O–C=O	12	0.04

[a] Lorentzian-Gaussian mixing ratio, [b] FWHM= Full width at half maximum.

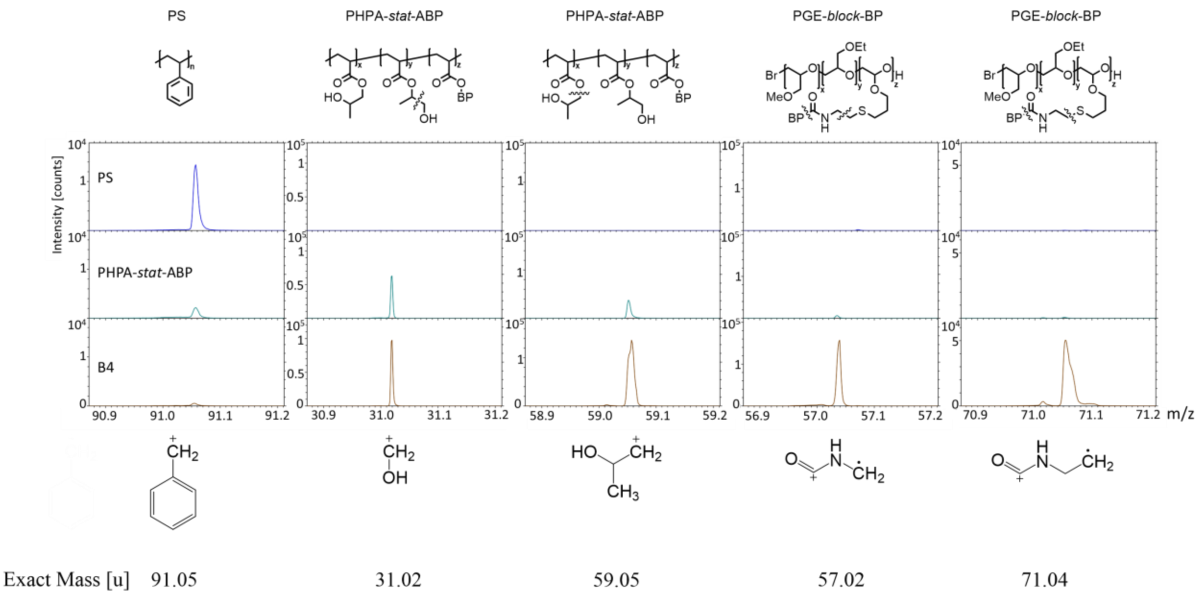

Figure S9. ToF-SIMS mass spectra of characteristic fragments obtained from 2D measurements on the basal PS layer, the PHPA-based gel layer **H**, and the bilayer **B4**.

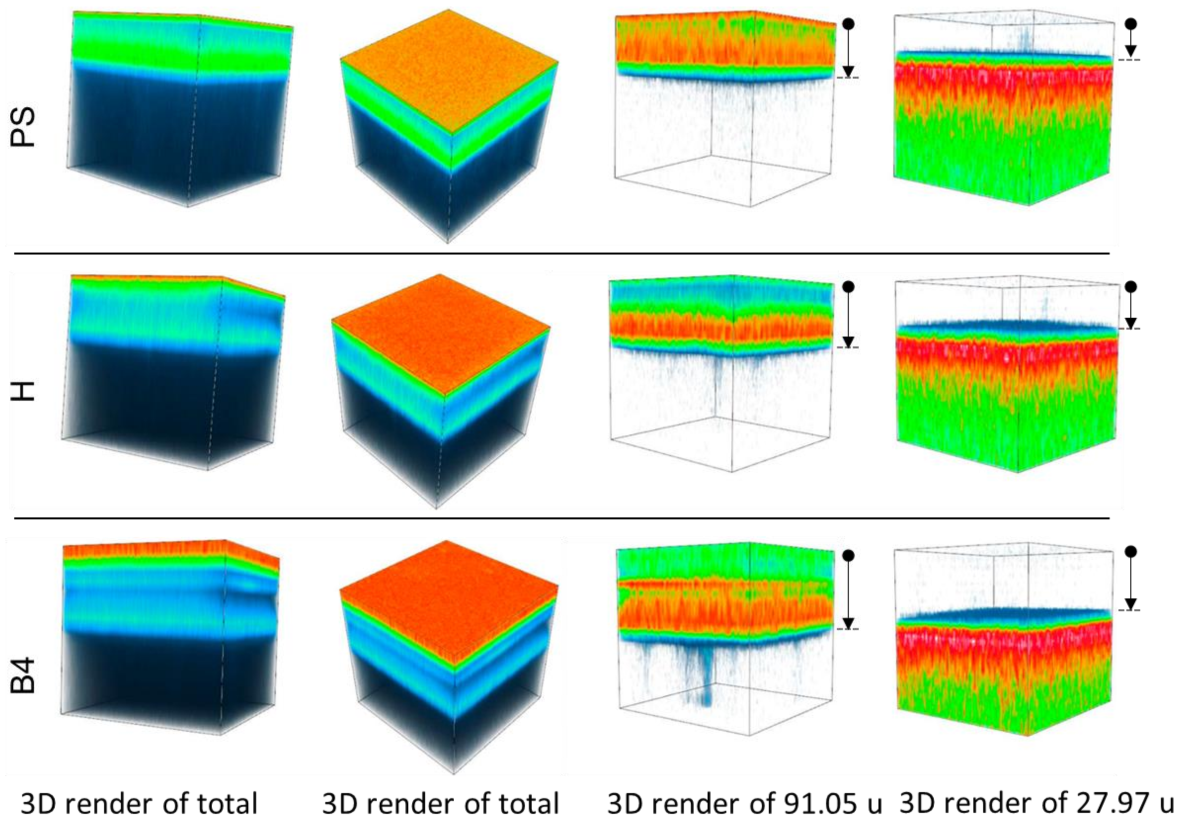

Figure S10. 3D rendered ToF-SIMS images of the total ion intensities (TC), the benzyl fragment $C_7H_7^+$ (91.05 u) and Si^+ (27.97 u) fragment at different tilt angles of the basal PS layer, the PHPA-based gel layer **H**, and the bilayer **B4**.

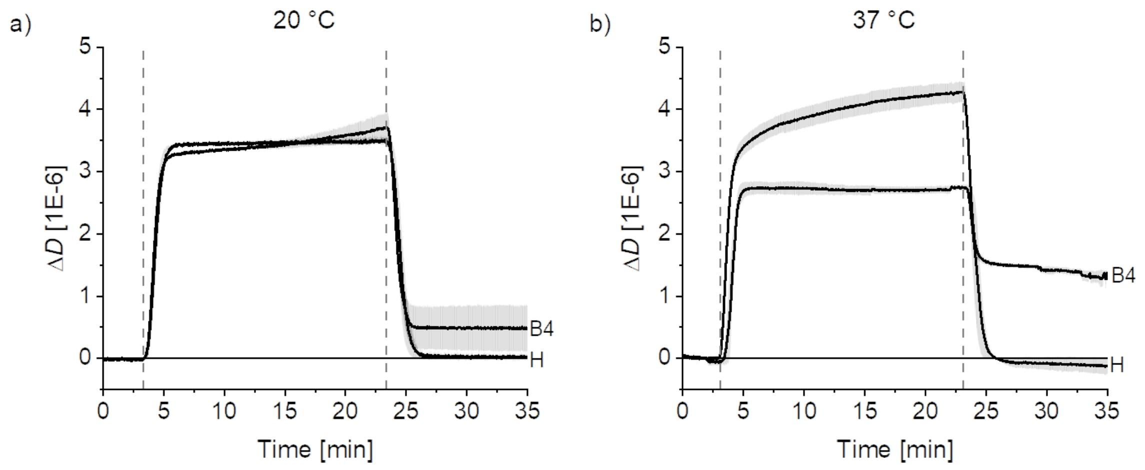

Figure S11. Complementary ΔD curves (3rd overtone) measured with QCM-D during the dynamic adsorption of proteins from FBS-supplemented cell culture medium (DMEM) on a PHPA-*stat*-ABP hydrogel **H** and on the corresponding brush-coated bilayer **B4** at **a)** 20 °C and **b)** 37 °C. Vertical dashed lines indicate an eluent change from PBS to DMEM and back to PBS. Data are plotted as average ± SD (gray shadow along the curves). (n = 3)

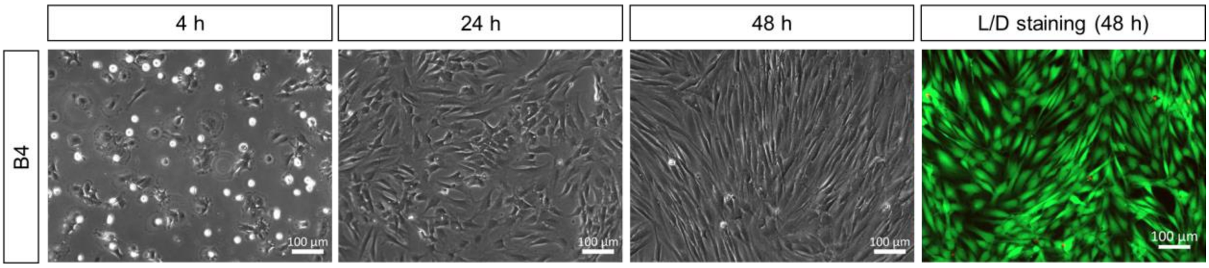

Figure S12. Representative phase contrast images of HDFs 4, 24, and 48 h after seeding on the **B4** bilayer coatings on Petri dishes as well as a fluorescence image of HDFs cultured for 48 h culture on **B4** after live/dead (L/D) staining with PI (red/D) and FDA (green/L). The dishes were not pre-incubated before cell seeding. Seeding density: 3.5×10^4 cells cm^{-2}. (n = 3)

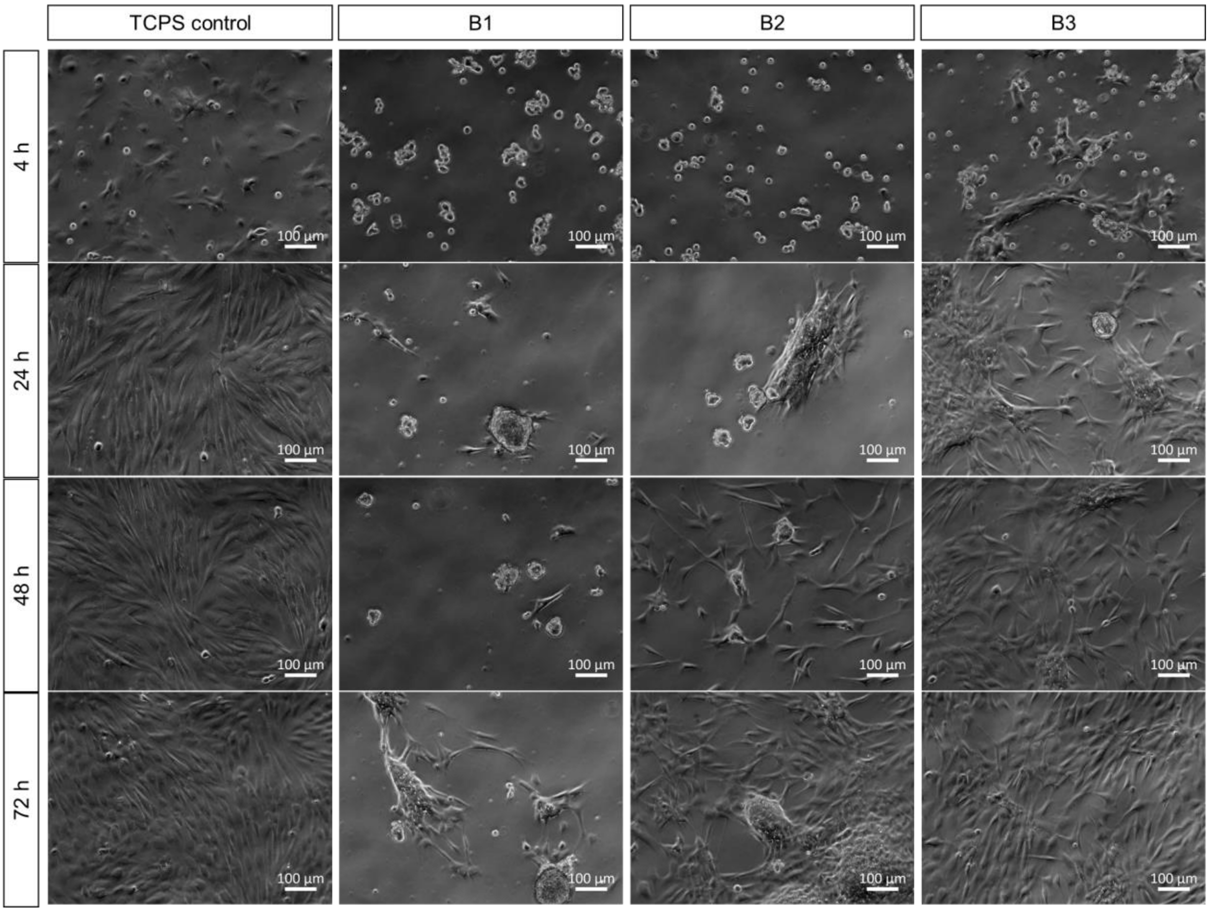

Figure S13. Representative phase contrast images of HDFs 4, 24, 48, and 72 h after seeding on the **B1, B2,** and **B3** bilayer coatings on Petri dishes. The dishes were pre-incubated in cell culture medium supplemented with 10% FBS for 30 min at 37 °C before cell seeding. Seeding density: 3.5×10^4 cells cm^{-2}. (n = 3)

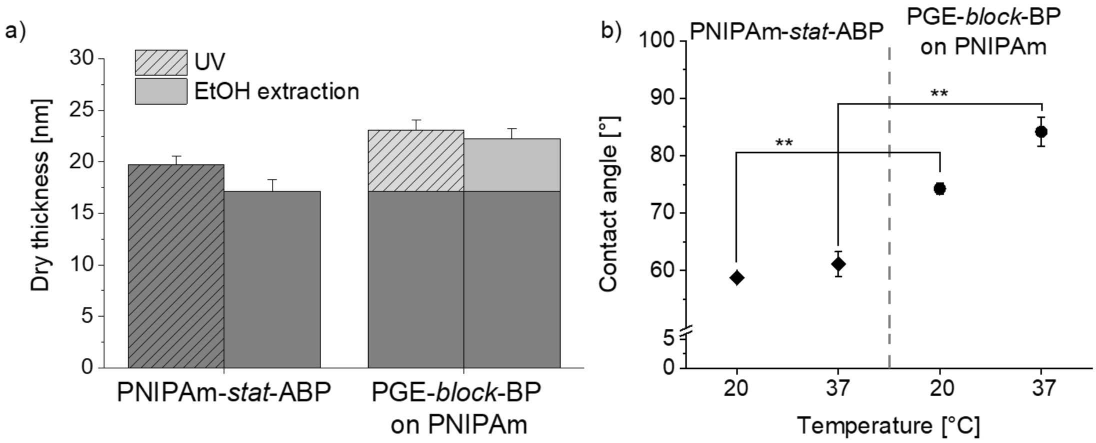

Figure S14. Thickness and wettability analysis of surface-bound PNIPAm-based hydrogels and brushed-up bilayers produced via the self-assembly of PGE-*block*-BP at a concentration of 250 µg mL^{-1} at RT (20 °C) for 1 h. **a)** Dry layer thickness after UV-immobilization (patterned) and extraction in ethanol (solid) determined on PS-coated silicon wafer substrates. **b)** Representative temperature-dependent static water contact angles of PNIPAm-*stat*-ABP (diamonds) measured right after water drop deposition and brushed-up PNIPAm coatings (circles) measured after 5 s of deposition. In **b)**, statistical significance was tested with a paired sample t-test (*, $p < 0.05$; **, $p < 0.005$). Normal distribution was assessed using the Shapiro-Wilk test ($p < 0.05$). Error bars indicate the standard deviation (SD). (n = 5)

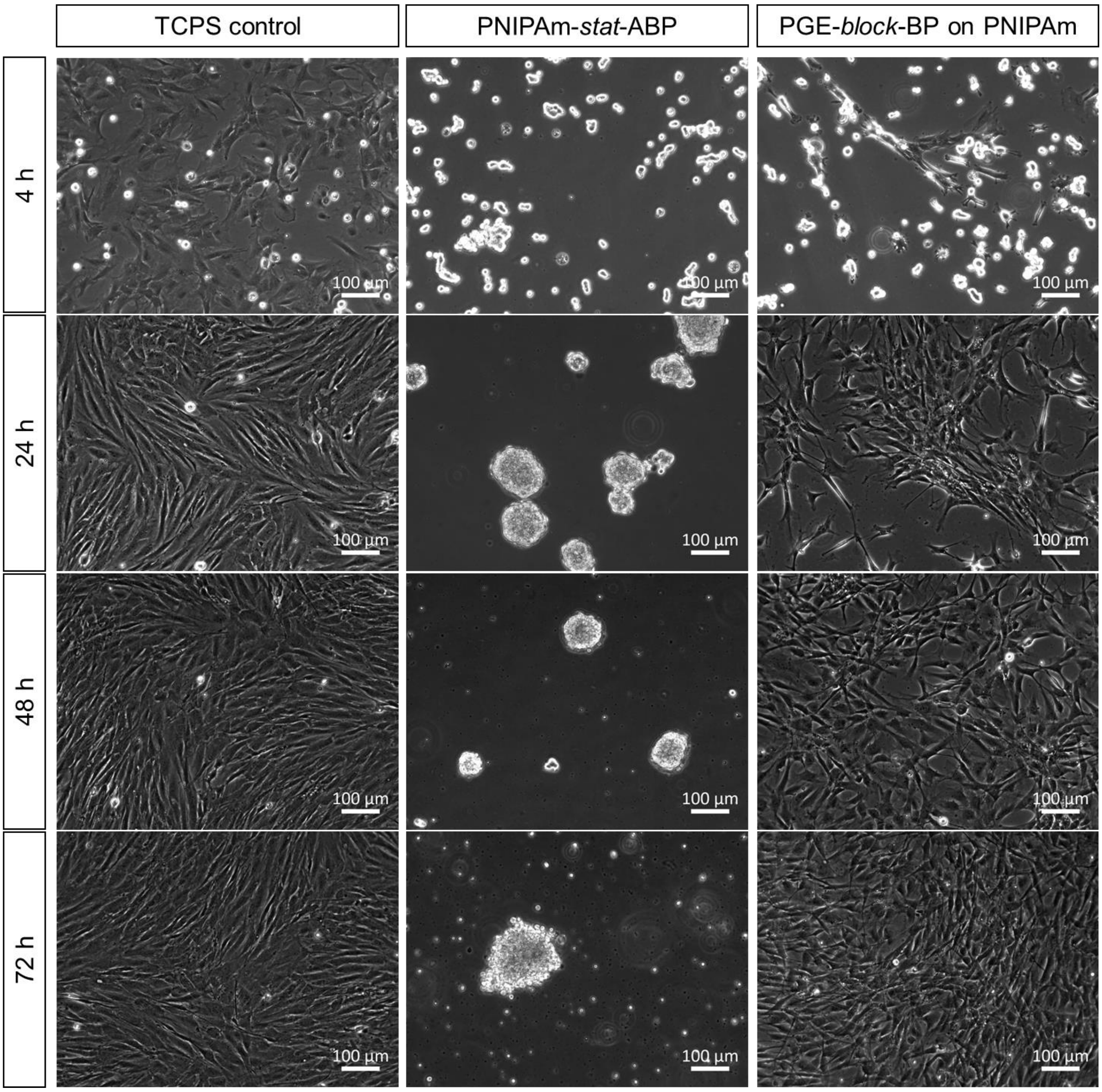

Figure S15. Representative phase contrast images of HDFs 4, 24, 48, and 72 h after seeding on TCPS control, PNIPAm-*stat*-ABP, and PGE-*block*-BP on PNIPAm-based gel coatings immobilized on Petri dishes. The dishes were pre-incubated in cell culture medium supplemented with 10% FBS for 30 min at 37 °C before cell seeding. Seeding density: 3.5 x 10^4 cells cm^{-2}. (n = 3)

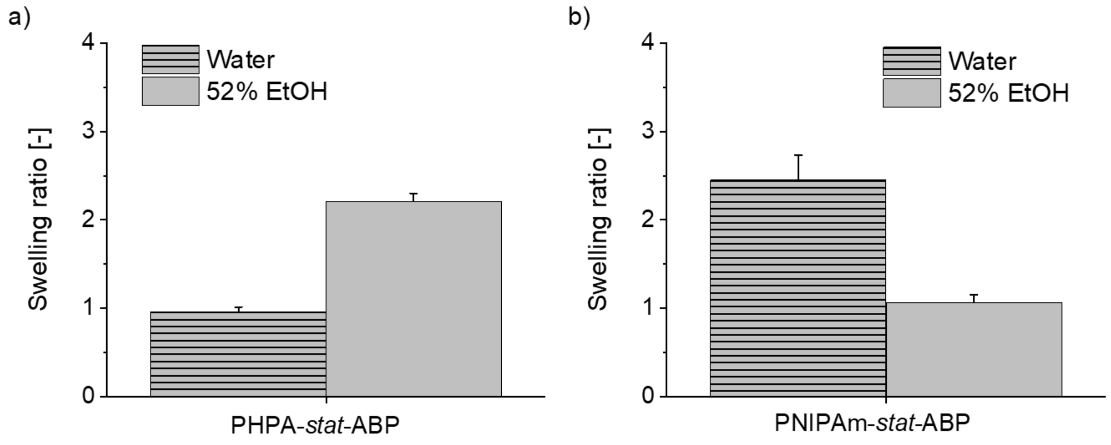

Figure S16. Swelling ratio of **a)** PHAP-*stat*-ABP and **b)** PNIPAm-*stat*-ABP hydrogel coatings in Milli-Q water (patterned) and in H_2O:EtOH (48:52 v/v-%) mixture (solid) measured at RT (20 °C) by SE. The swelling ratio (SR) was calculated according to $SR = (d_{wet} - d_{dry})/d_{dry}$ where d_{wet} and d_{dry} correspond to the thickness of the equilibrated wet and gel coatings. Both gels are in a well-solvated state in the selective solvent. Error bars indicate the standard deviation. (n = 3)

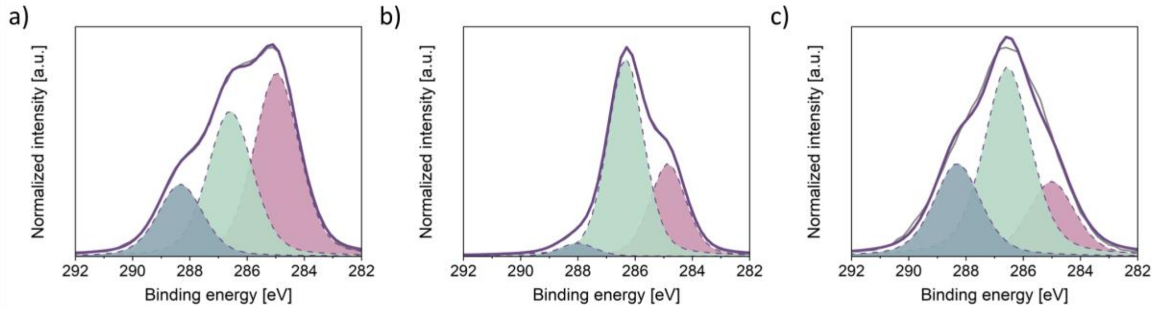

Figure S17. Highly resolved C1s XP spectra (gray line) of the **a)** alginate coating on a silicon wafer, **b)** PGE-functionalized alginate, and **c)** UV-irradiated alginate coating as control. Fitted peak components and the sum curve of all the fitted peak components are illustrated by the purple dashed and solid lines, respectively.

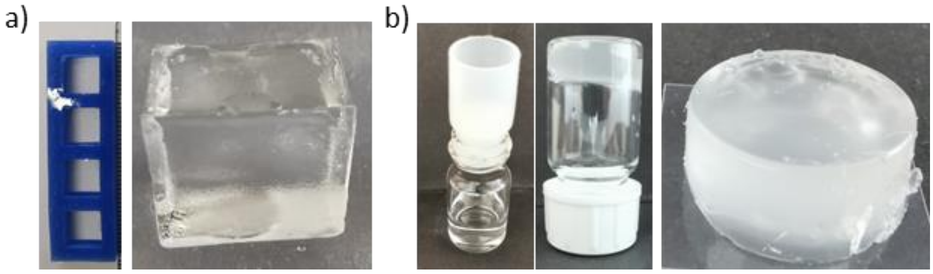

Figure S18. Macroscopic photographs of **a)** a photo-crosslinked PHPA-*stat*-ABP bulk gel produced by UV-irradiation of a PHPA-*stat*-ABP solution (500 mg mL^{-1}) in ethanol for 26 min (2 x 13 min) in a self-casted blue silicone mask (7 mm x 7 mm x 4 mm) shown on the left, and **b)** a septum-sealed vial (⌀ = 20 mm) with the monomer solution and flip test showing gelled thermally-crosslinked PHPA prepared from HPA with *N*,*N*'-methylene bisacrylamide (MBAA) as a crosslinker in MeOH through free radical polymerization in the presence of AIBN as initiator and resulting PHPA-MBAA bulk gel shown on the right.

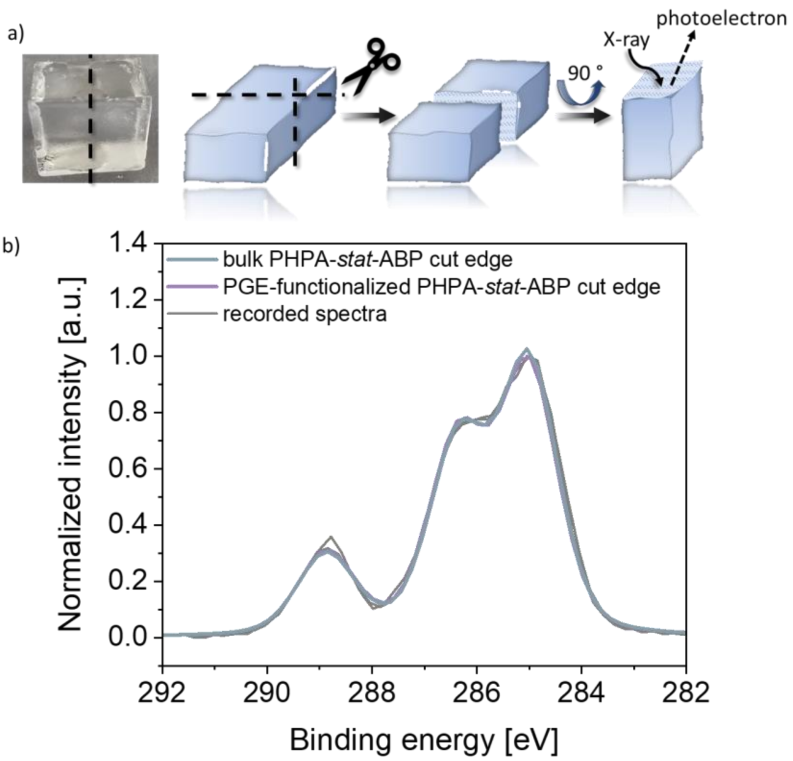

Figure S19. a) Illustration of the "cutting-edge" method employed for XPS analysis of the non-functionalized as well as the PGE-functionalized PHPA-*stat*-ABP bulk gel and **b)** sum curves of fitted background subtracted and normalized highly-resolved C1s XP spectra of the surfaces of freshly cut edges of a non-functionalized (blue) and PGE-functionalized PHPA-*stat*-ABP bulk gel (purple). Both spectra are identical and do not indicate any penetration of PGE copolymers into the bulk gel interior during the self-assembly process. Raw background subtracted and normalized highly resolved C1s XP spectra are depicted in gray.

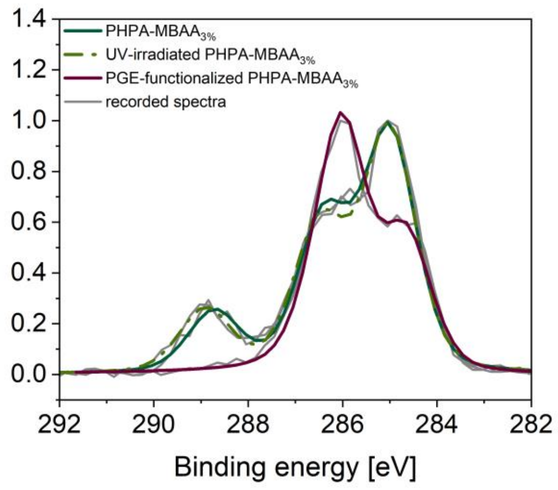

Figure S20. Fitted and non-fitted background subtracted and normalized highly resolved C1s XP spectra of the pristine, non-functionalized PHPA-MBAA bulk gel and the PGE-functionalized PHPA-MBAA bulk gel as well as the UV-irradiated non-functionalized gel as control. UV irradiation did not significantly affect the composition of the surface of the bulk PHPA-MBAA gel, while PGE-brush modification effectively suppressed the C-CO-O signal components of the basal gel substrate, indicating a PGE brush thickness of $\geq$ 10 nm.

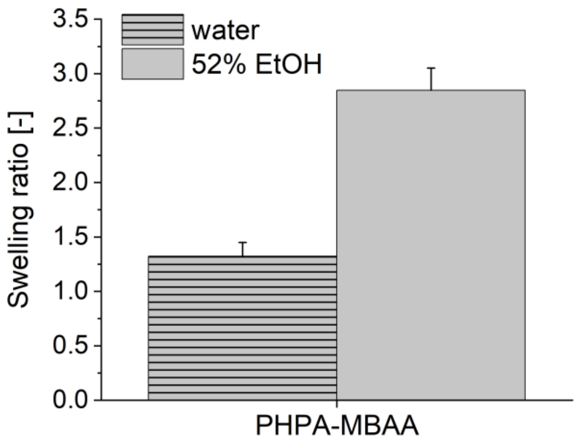

Figure S21. Swelling ratio of PHPA-MBAA bulk gels in Milli-Q water (patterned) and aqueous ethanol (48 v/v-% H_2O) (solid) after 90 min equilibration. Error bars indicate the standard deviation. The bulk gels are swollen in both solutions. (n = 3)

REFERENCES

(1) Stöbener, D. D.; Weinhart, M. Thermoresponsive Poly(Glycidyl Ether) Brush Coatings on Various Tissue Culture Substrates-How Block Copolymer Design and Substrate Material Govern Self-Assembly and Phase Transition. *Polymers (Basel)* **2020**, *12(9)* (9), 1899. DOI: 10.3390/polym12091899.
(2) Stöbener, D. D.; Hoppensack, A.; Scholz, J.; Weinhart, M. Endothelial, Smooth Muscle and Fibroblast Cell Sheet Fabrication from Self-Assembled Thermoresponsive Poly(Glycidyl Ether) Brushes. *Soft Matter* **2018**, *14* (41), 8333-8343. DOI: 10.1039/c8sm01099d.
(3) Eliassi, A.; Modarress, H.; Mansoori, G. A. Densities of Poly(Ethylene Glycol) + Water Mixtures in the 298.15–328.15 K Temperature Range. *J. Chem. Eng. Data* **1998**, *43* (5), 719-721. DOI: 10.1021/je970228a.
(4) Hu, Y.; Jin, J.; Han, Y.; Yin, J.; Jiang, W.; Liang, H. Study of Fibrinogen Adsorption on Poly(Ethylene Glycol)-Modified Surfaces Using a Quartz Crystal Microbalance with Dissipation and a Dual Polarization Interferometry. *RSC Adv.* **2014**, *4* (15), 7716-7724, 10.1039/C3RA46934D. DOI: 10.1039/C3RA46934D.
(5) Kim, M.; Schmitt, S. K.; Choi, J. W.; Krutty, J. D.; Gopalan, P. From Self-Assembled Monolayers to Coatings: Advances in the Synthesis and Nanobio Applications of Polymer Brushes. *Polymers* **2015**, *7* (7), 1346-1378. DOI: 10.3390/polym7071346.
(6) Heinen, S.; Cuéllar-Camacho, J. L.; Weinhart, M. Thermoresponsive Poly(Glycidyl Ether) Brushes on Gold: Surface Engineering Parameters and Their Implication for Cell Sheet Fabrication. *Acta Biomater.* **2017**, *59*, 117-128. DOI: 10.1016/j.actbio.2017.06.029 From NLM.
(7) Lee, H.; Venable, R. M.; MacKerell, A. D.; Pastor, R. W. Molecular Dynamics Studies of Polyethylene Oxide and Polyethylene Glycol: Hydrodynamic Radius and Shape Anisotropy. *Biophys. J.* **2008**, *95* (4), 1590-1599. DOI: 10.1529/biophysj.108.133025.

(8) Heinen, S.; Weinhart, M. Poly(Glycidyl Ether)-Based Monolayers on Gold Surfaces: Control of Grafting Density and Chain Conformation by Grafting Procedure, Surface Anchor, and Molecular Weight. *Langmuir* **2017**, *33* (9), 2076-2086. DOI: 10.1021/acs.langmuir.6b03927.
(9) Stöbener, D. D.; Weinhart, M. On the Foundation of Thermal "Switching": The Culture Substrate Governs the Phase Transition Mechanism of Thermoresponsive Brushes and Their Performance in Cell Sheet Fabrication. *Acta Biomater.* **2021**, *136*, 243-253. DOI: 10.1016/j.actbio.2021.09.012.
